# Computational Screening Guiding the Development of a Covalent-Organic Framework-Based Gas Sensor for Early Detection of Lithium-Ion Battery Electrolyte Leakage

**DOI:** 10.1021/acsami.4c19321

**Published:** 2025-02-03

**Authors:** Liangdan Zhao, Chunyi Yu, Xiaoyu Wu, Mingrui Zuo, Qian Zhang, Qiuchen Dong, Lifeng Ding

**Affiliations:** † Department of Chemistry and Materials Science, Advanced Materials Research Center, School of Science, Xi’an Jiaotong-Liverpool University, Suzhou, Jiangsu, 215123, P. R. China; ‡ Department of Chemistry and Materials Innovation Factory, University of Liverpool, Liverpool L69 7ZD, U.K.; § Department of Chemical and Biomolecular Engineering, National University of Singapore, Singapore 117585, Singapore

**Keywords:** covalent-organic framework (COF), ethylene carbonate, GCMC simulation, lithium-ion battery, chemiresistive gas sensor, DFT calculation

## Abstract

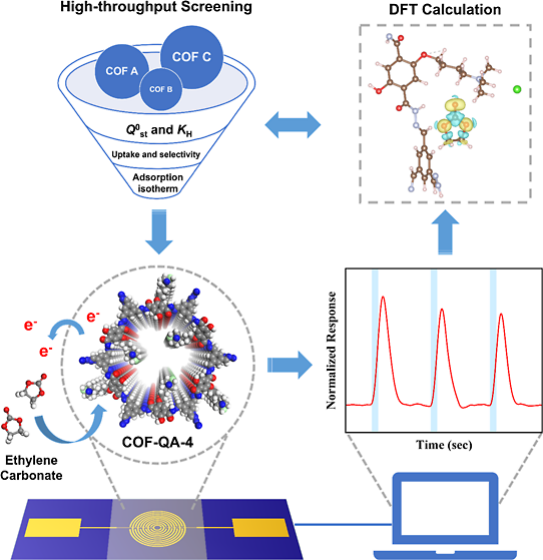

This study presents a computationally guided approach for selecting covalent organic frameworks (COFs) for the selective detection of the trace ethylene carbonate (EC) vapor, a key indicator of electrolyte leakage from lithium-ion batteries (LIBs). High-throughput screening, employing grand canonical Monte Carlo (GCMC) simulation complemented by density functional theory (DFT) calculations, was used to identify potential COF candidates from the CURATED COF database. Among the screened materials, an imine COF functionalized with quaternary ammonium (QA) groups, named COF-QA-4, exhibited a high adsorption capacity (5.88 mmol/g) and selectivity of EC vapor. DFT analysis revealed strong molecular interactions driven by a partial charge transfer mechanism between EC and the COF-QA-4 framework, underpinning its superior adsorption properties. Experimental validation through chemiresistive gas sensors fabricated with COF-QA-4 demonstrated excellent sensitivity and reversibility to 1.15 ppmv of EC vapor, maintaining consistent performance over three response–recovery cycles. This work highlights the potential of computationally guided material discovery for advancing sensor technologies in LIB safety monitoring.

## 1 Introduction

Lithium-ion batteries (LIBs) stand among the forefront power sources for portable electronic devices (e.g., smartphones, laptops, and flashlights), electric vehicles, military applications, and aerospace domains owing to their exceptional attributes, including high energy density, minimal environmental impact, consistent performance, and long lifespan.^1−3^ Despite ongoing efforts to enhance energy density and lower costs, LIB failures caused by external damage (such as heating, extrusion, collision, overcharging, etc.) or possible defects inside the cell (such as lithium dendrites) pose a threat to the safe use of LIB, which should raise the attention of human beings. In 2021 alone, incidents involving LIB failures led to over 20 instances of fire or explosion in electric vehicles worldwide.^4^ The flammability in LIBs stems from the solvents that serve as electrolytes. Presently, predominant among commercially available solvents used in LIBs are organic carbonates (OCs), such as ethylene carbonate (EC) and propylene carbonate (PC). The low flash points of these OC vapors raise significant safety concerns regarding their flammability and potential for explosion. During the thermal runaway (TR) stage of LIB failures, the decomposition of electrolytes can rapidly increase the temperature to 200–300 °C.^5^ When the battery cells are damaged and come into contact with air, the vented electrolyte OCs gases will ignite, eventually leading to battery fire or explosion. Therefore, early detection of electrolyte leakage and the prevention of extensive damage are of vital importance, and the trace OC vapor is an attractive tracer gas for LIB failure detection before the TR.

It is recognized that currently, no widely employed techniques exist within portable electronic devices and electric vehicles for the early detection of OC gases of LIB electrolyte leakage. Consequently, there is an urgent imperative to develop gas sensors explicitly tailored for the early detection of leaked OC gases at room temperature. To detect leakage in portable electronic devices or electric vehicles, gas sensors should be low-cost, embeddable, long-life, instant, sensitive, and selective to target gases. Recently, the widely used gas sensor based on the metal oxide semiconductor (MOS) has the potential for the effective and rapid detection of analytes. Still, they lack gas selectivity and are mostly found to be sensitive at high temperatures. Nondispersive infrared (NDIR) gas sensors have attractive characteristics, including superior selectivity and long life.^6^ However, the difficulty of miniaturization, the complexity of configuration, and the high cost limit their large-scale application. Therefore, this has invited research efforts to develop alternative gas sensors with excellent sensing performance in sensitivity and selectivity and reduced size, cost, and power consumption. Combining the current micromachining technology with new nanoporous materials has been suggested as a promising approach to solve the challenges.

Covalent organic frameworks (COFs) are a kind of novel nanoporous materials self-assembled by strong covalent bonds (such as B–O, C–N, C=N, and C=C–N) between organic building blocks composed of the light elements (such as B, C, Si, N, and O).^7,8^ They have the properties of tunable pore structures, large internal surface areas, adjustable chemical functionalities, and reversible adsorption, making them promising candidate materials for the selective recognition and sensing of specific gas species, especially volatile organic compounds (VOCs). Since Yaghi and co-workers synthesized the first COF in 2005, more than 800 COFs have been reported and employed in various applications, including gas storage/separation, optoelectronics, catalysis, and chemo-/biosensing.^9,10^ Most COFs and other pure organic conjugated materials have low intrinsic conductivity due to the relatively large energy gap (≈2 eV).^11^ In order to achieve conductivity, the synthesized COFs need to be activated, which involves removing the electrons in the valence band and/or adding electrons to the conduction band. The activation methods include thermal excitation, photoexcitation, and doping, and doping is the most efficient way to produce a large number of charge carriers.^12^ Attributed to the latest progress in the structural design of COFs, chemiresistive gas sensors based on conductive COFs have become possible and have already been used to detect various polluting gases such as benzene,^6^ NH_3_,^13,14^ and H_2_S.^13^ However, few examples of OC gas detection have been reported. Hence, developing a COF-based gas sensor for selective detection of OCs and VOCs using the electrical transduction principle is a promising topic in the future.

**Figure 1:**
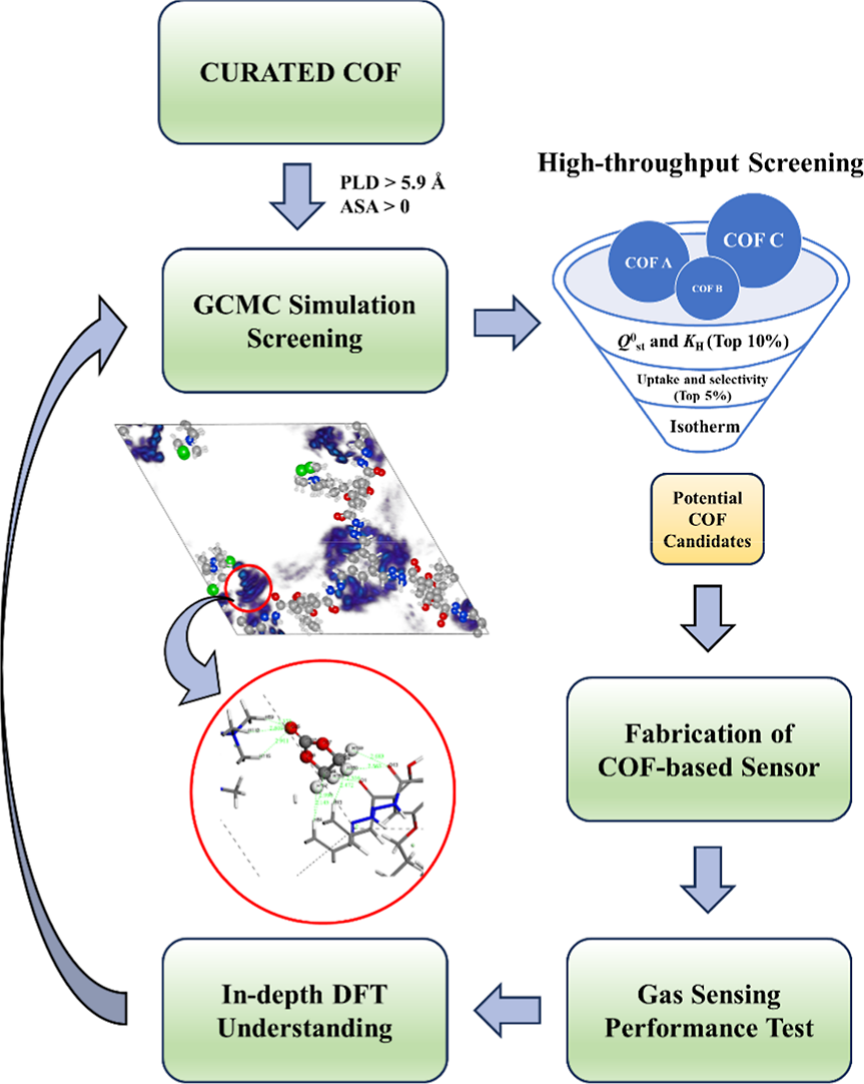
Protocol of the COF screening process for the EC selective adsorption using a computational-aided method.

In this work, we employ a two-step approach combining computational screening and practical gas-sensing experiments to identify and validate a suitable COF for the selective detection of EC, a representative OC leaked from LIBs. The protocol of the COF screening process is shown in Figure 1. Given the heavy computational resources associated with density functional theory (DFT) calculations, high-throughput screening (HTS), employing grand canonical Monte Carlo (GCMC) simulation, was initially applied to narrow down the range of promising COF candidates with high potential for selectively adsorbing trace EC vapor in Clean, Uniform, and Refined with Automatic Tracking from Experimental Database (CURATED) of COFs. Three stages were used to screen COFs that could selectively adsorb trace EC vapor against other gas impurities.

Stage 1: COFs with *Q*^0^_st_ and *K*_H_ are both in the top 10%.

Stage 2: COFs with EC uptake and selectivity are both in the top 50%.

Stage 3: (1) COFs with linear increase range of adsorption isotherm at low EC gas concentrations, (2) COFs with good selectivity of EC against other gas impurities, and (3) COFs with electrical conductivity at room temperature.

An imine-based COF, COF-QA-4, emerged as a top candidate for EC vapor capture in HTS, demonstrating a high adsorption capacity of 5.88 mmol/g and exceptional selectivity toward EC. DFT calculations further elucidated the interaction mechanism between the EC molecules and the COF-QA-4 framework, revealing that adsorption is facilitated by partial charge transfer. COF-QA-4 was subsequently synthesized via a Schiff-base reaction between 1,3,5-triformylbenzene (TFB) and 2-hydroxy-5-(trimethylammonio-butoxy)-terephthalohydrazide (QA-4), following the method reported by He et al.^15^ Chemiresistive sensing performance tests demonstrated that sensors fabricated with COF-QA-4 exhibited excellent responsiveness to 1.15 ppmv EC vapor, achieving stable detection within 120 s of exposure. This work represents the first COF-based gas sensor for EC detection, establishing a new framework for the rational design of gas sensors tailored to specific target molecules by integrating in silico screening with experimental validation.

## 2 Methodology

### 2.1 COF Database Preparation and GCMC Simulation Details

612 COFs from the CURATED COF database were selected for high-throughput GCMC calculations to screen optimum COF candidates for EC sensing.^16^ All of the COFs’ structure files are solvent-free and disorder-free, suitable for direct use as input files for computational screening purposes. The geometric properties of COFs, such as pore-limiting diameter (PLD) and accessible surface area (ASA), were computed using the Zeo++ software package^17,18^ with a probe of N_2_ spherical model of radius 1.86 Å.^18,19^ Gaussian 09 package was used to calculate initial geometry optimizations and atomic charges of the EC all atoms (AA) model using the B3LYP method combined with the 6-31G(d,p) basis set (Table S1).^20^ The minimum molecular dimension of an EC molecule was calculated as 5.9 Å (Figure S1), which means that the EC molecule can enter a COF with a PLD greater than 5.9 Å. Before the GCMC simulations screening of the CURATED, COFs with PLD <5.9 Å and ASA = 0 m^2^/g were excluded, and the candidates of COF narrowed down to 564 (Figure S2).

The adsorption behavior of EC molecules in the COFs at room temperature (298 K) under atmospheric pressure (1 bar) was calculated by GCMC simulations using the RASPA software package.^21−23^ The EC molecules and COF frameworks were considered to be rigid in the GCMC simulations. The nonbonding intermolecular interactions among gas molecules, as well as between gas molecules and the COFs, were addressed employing Lennard-Jones (LJ) and Coulombic potentials
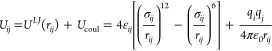
where *r*_*ij*_, ε_*ij*_, and σ_*ij*_ are the atom–atom distance, the LJ well depth, and the equilibrium distance for the interactions between atoms *i* and *j*, respectively.^24,25^ For LJ interactions, a cutoff distance of 12 Å was applied. The force field parameters for COF framework atoms were adopted from the universal force-field^26^ and DREIDING force-field.^27,28^ The optimized potentials for liquid simulations (OPLS)^29^ and transferable potentials for phase equilibria(TraPPE) force fields^30^ were utilized for modeling EC molecules. To ascertain the most suitable force field for describing the interactions between the framework and EC guest molecules, various combinations of the force fields, as mentioned earlier, were explored in GCMC simulations (Tables S2 and S3). The force field parameters for other impurity gas molecules (including CO, CO_2_, O_2_, H_2_, methane, ethene, and ethane) were collected from the previously reported literature (Table S4). The Jorgensen mixing rule was applied to mix LJ parameters for different interaction pairs. The partial charges of COF framework atoms were acquired by the density-derived electrostatic and chemical (DDEC) method.^31^ Electrostatic calculations were performed by employing the Ewald summation method. Each GCMC simulation comprised 200,000 cycles, with the initial 100,000 cycles for equilibration and the subsequent 100,000 cycles for ensemble averaging. Within each cycle, *n* trial moves (where *n* represents the number of adsorbate molecules), including translation, rotation, reinsertion, and swap operations (insertion and deletion) were executed.^32^ The helium void fraction (HVF), Henry’s adsorption constant (*K*_H_), and isosteric heat of adsorption at zero coverage (*Q*^0^_st_) were also calculated using the Widom insertion method using RASPA software.^33^

By conducting GCMC simulation calculations on the adsorption performance of three components (1 ppmv EC, 1 ppmv impurity, and the remaining component is N_2_) mixtures in candidate COFs, the adsorption amounts of EC, impurity, and N_2_ in COFs can be obtained at 298 K and 1 bar. The selectivity of EC against impurity (*S*_EC/Impurity_) can be obtained by the following equation
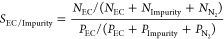
where *N* and *P* are the uptake and partial pressure of gases, respectively.

### 2.2 DFT Calculation Details

The first-principles calculations were performed utilizing the Vienna Ab initio Simulation Package (VASP) software package^34−37^ using the DFT. The Perdew–Burke–Ernzerhof form of the generalized gradient approximation (GGA-PBE) was employed to calculate the total energy and perform structural relaxations.^38^ Electron–ion interactions were evaluated using the projector-augmented wave (PAW) method.^39,40^ A dispersion correction with Becke-Johnson (BJ) damping (DFT-D3(BJ)) was used to incorporate the long-range van der Waals interactions.^41,42^ A plane-wave expansion with a cutoff energy of 600 eV was utilized, along with the Gaussian smearing method employing a width of 0.05 eV for all calculations. Brillouin zone integrations were implemented using the Γ-centered Monkhorst–Pack (MP) method to generate a *k*-point grid, where 1 × 1 × 3 was used for the COF bulk structure.^43^ The calculated results were obtained under sufficient convergence criteria for the total energy (10^–4^ eV) and force (0.01 eV/Å), respectively. The adsorption energy of the gas molecule in the COF was calculated by the following equation
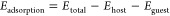
where *E*_host_ and *E*_guest_ are energies of the host framework and the free gas molecules, respectively, and the *E*_total_ is the single-point energy of the optimized binding complex of the adsorbate and framework. The bulk model of the COF experienced geometry optimization before calculating the *E*_total_. Furthermore, the charge transfer, charge density difference (CDD), and electron localization function (ELF) were calculated using Bader charge analysis to analyze the charge transfer behavior upon the adsorption.^43^ The density of state (DOS) and band structure were also calculated to further demonstrate the electronic structure of the COF. The high-symmetry *k*-points coordinates of COF were computed using the SeeK-path tool.^44,45^

## 3 Results and Discussion

### 3.1 GCMC Screening of EC Gas Adsorption in COFs

**Figure 2:**
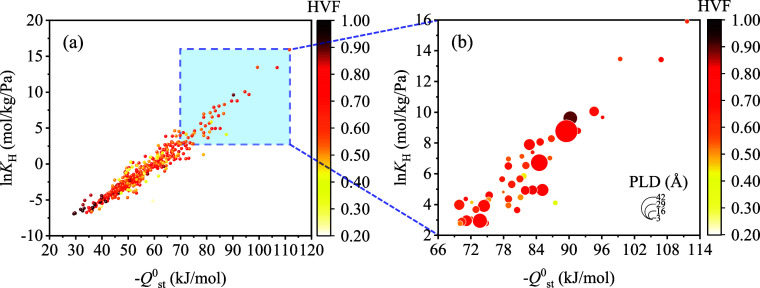
(a) –*Q*^0^_st_ versus ln*K*_H_ of EC adsorption in selected 564 COFs in stage 0. (b) *–Q*^0^_st_ versus ln*K*_H_ of EC adsorption in 46 COFs in stage 1. The dot size in (b) represents the PLD of the COFs.

Henry adsorption constant (*K*_H_) and isosteric heat of adsorption at zero coverage (*Q*^0^_st_) indicate the strong binding ability of the framework with guest molecules in the very low pressure (Henry’s law) region, which means the preference for adsorption of gas molecules in the adsorbent.^46,47^ In stage 1, the Henry adsorption constant (*K*_H_) and isosteric heat of adsorption at zero coverage (*Q*^0^_st_) of EC were obtained using Widom’s insertion using RASPA software. Figure 2a shows the screening results of *–Q*^0^_st_ versus ln*K*_H_ of EC in 564 COFs. COFs with both –*Q*^0^_st_ and *K*_H_ of EC ranked in the top 10% initially remained for the next screening stage. 46 COFs were initially selected in stage 1 with the *Q*^0^_st_ range from –69.87 to –111.64 kJ/mol and *K*_H_ range from 15.94 to 8.08 × 10^6^ mol/kg/Pa (Figure 2b).

**Figure 3:**
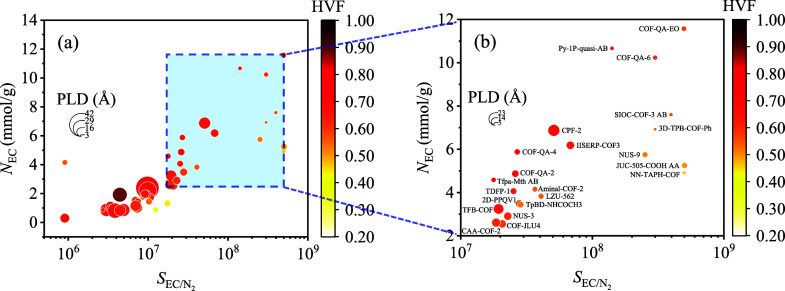
(a) EC uptake versus selectivity of EC against N_2_ in selected 46 COFs in stage 1. (b) EC uptake versus EC selectivity against N_2_ in 22 COFs selected in stage 2.

In stage 2, the uptake of 1 ppmv EC vapor in 46 COFs at 298 K and 1 bar was calculated by GCMC simulation. There was 1 ppmv EC vapor in the system, and the remaining component was balance gas N_2_. Figure 3a shows the GCMC screening results of EC uptake (*N*_EC_) versus selectivity of EC against N_2_ () in 46 COFs. COFs with both EC uptake and selectivity ranking in the top 50% of COFs remained for the next screening stage. Twenty-two COFs with EC uptake range from 2.55 to 11.57 mmol/g and selectivity range from 1.78 × 10^7^ to 5.03 × 10^8^ were selected in stage 2 (Figure 3b).

**Figure 4:**
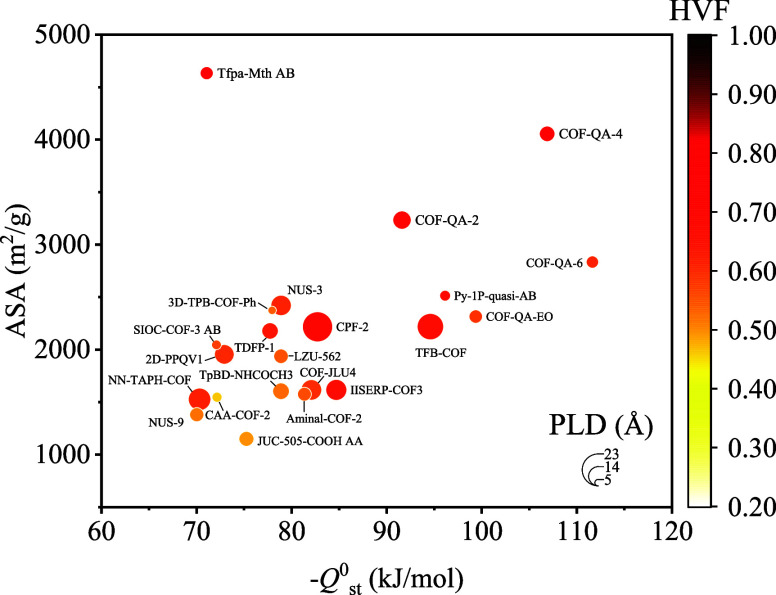
–*Q*^0^_st_ of EC in 22 COFs and pore properties of 22 COFs that were selected in stage 2.

In the second stage, it was observed that among the COFs selected, those with PLD ranging from 5.98 to 21.34 Å, ASA ranging from 1149.92 to 4633.23 m^2^/g, HVF ranging from 0.461 to 0.738, and *Q*^0^_st_ ranging from –111.64 to –70.03 kJ/mol exhibited notable selectivity for adsorbing trace EC gas over N_2_, as illustrated in Figure 4.

In stage 2, only EC and N_2_ were considered in the system for the GCMC calculation to reduce the number of COF candidates quickly. However, the EC detection performance of COFs in the case of other impurity gases in LIBs electrolyte leakage should also be considered. In the third stage, the EC adsorption selectivities against seven impurity gases (CO, CO_2_, O_2_, H_2_, methane, ethene, and ethane) in 22 COFs were computed, as depicted in Figure S3. During the simulations, both impurity gases and EC were maintained at concentrations of 1 ppmv, with nitrogen (N_2_) constituting the remaining gas component up to 1 bar pressure. A higher *S*_EC_ value indicates heightened EC selectivity, implying that EC is preferentially adsorbed and detected by COFs in the presence of impurity gases. Finally, 12 COFs, namely, CPF-2, NUS-3, IISERP–COF3, SIOC–COF-3 AB, NN-TAPH–COF, JUC-505–COOH AA, COF–QA-2, COF–QA-4, COF–QA–EO, 2D-PPQV1, LZU-562, and 3D-TPB–COF–Ph with lower selectivities of seven gas impurities were labeled as high-performance COFs for EC selective detection. Notably, following our hierarchical screening approach made it possible to downsize the number of COFs to only 2% of the initial database, which is subsequently employed in our screening study’s computationally most demanding stage.

**Figure 5:**
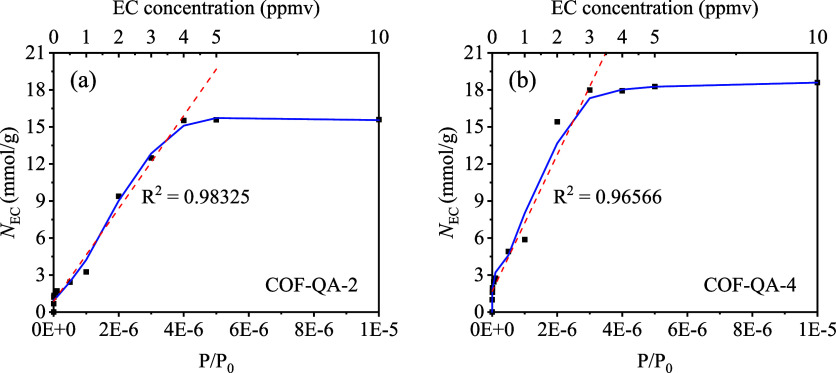
EC adsorption isotherms of (a) COF-QA-2 and (b) COF-QA-4 under 1 bar pressure with N_2_ at 298 K calculated from GCMC simulations.

The EC adsorption isotherms of 12 COFs were also calculated **(**Figures 5 and S4). Particularly, it can be seen that COF-QA-2 and COF-QA-4 had linear relationships between partial pressure and adsorption capacity at infinite dilution (Henry’s law region of adsorption). At last, COF-QA-4 was selected for the synthesis and sensing performance test because of its highest –*Q*^0^_st_ value and best EC selectivity among the abovementioned COFs.

**Figure 6:**
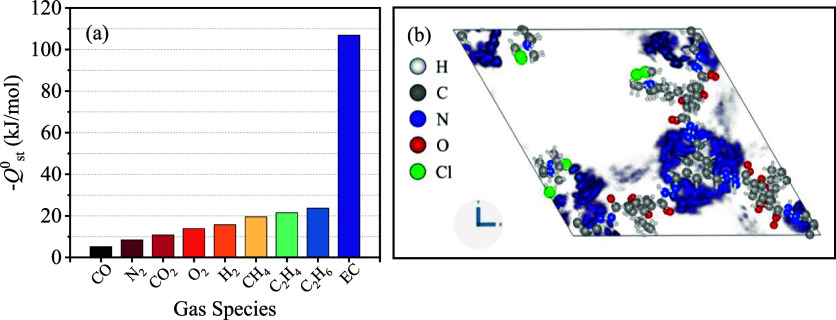
(a) –*Q*^0^_st_ of EC, and impurity gases in COF-QA-4. (b) Adsorption density plot of 1ppmv of EC in COF-QA-4 at 298 K.

The –*Q*^0^_st_ values of EC, N_2_, and impurity gases (CO, CO_2_, O_2_, H_2_, methane, ethene, and ethane) in COF-QA-4 were calculated and are depicted in Figure 6a. The results evidenced that COF-QA-4 had a stronger affinity of EC against other gas impurities. The adsorption density plot of 1 ppmv of EC in COF-QA-4 at 298 K is shown in Figure 6b.

### 3.2 DFT Calculations of the Sensing Mechanism

**Figure 7:**
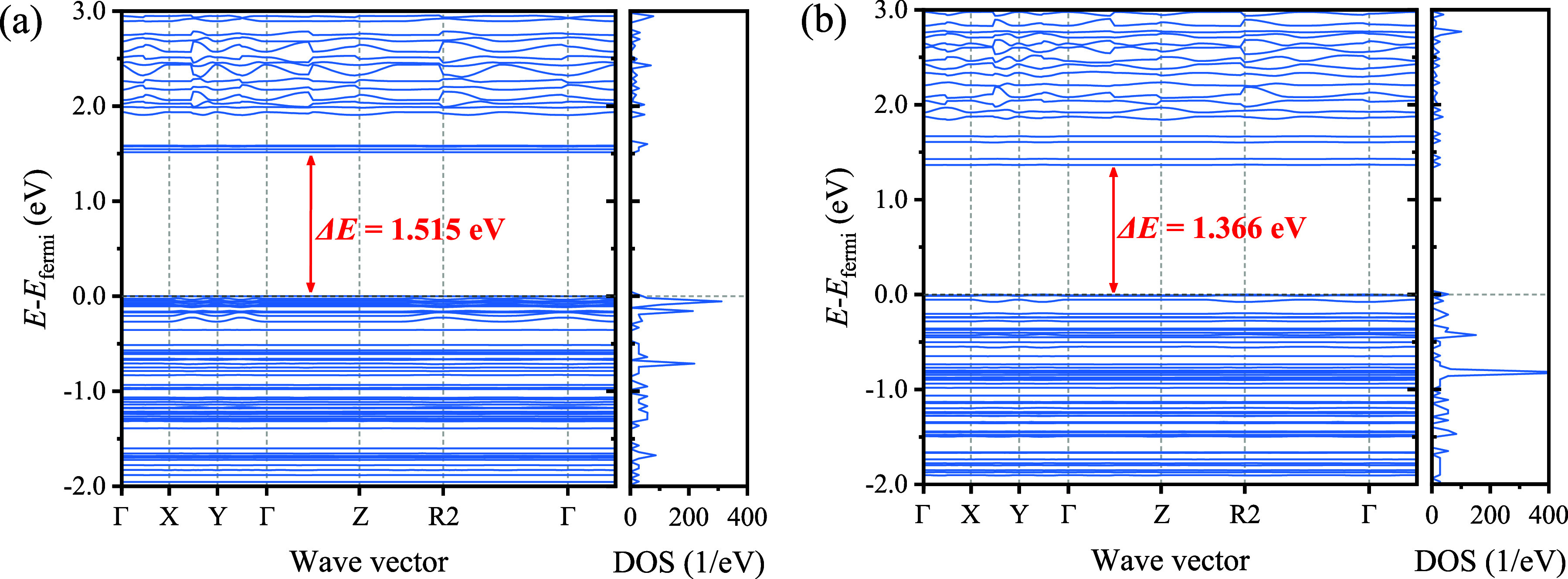
Band structure and density of state (DOS) of COF-QA-4 (a) before and (b) after EC adsorption.

DFT calculations were employed to further understand the sensing mechanism of the EC adsorption. First, the electronic structure of COF-QA-4 was obtained after the geometry optimization (Figure 7a). According to the filling of the electronic states in the calculated band structure, COF-QA-4 is a p-type semiconductor with a band gap of 1.515 eV and mainly relies on holes for electrical conduction.

**Figure 8:**
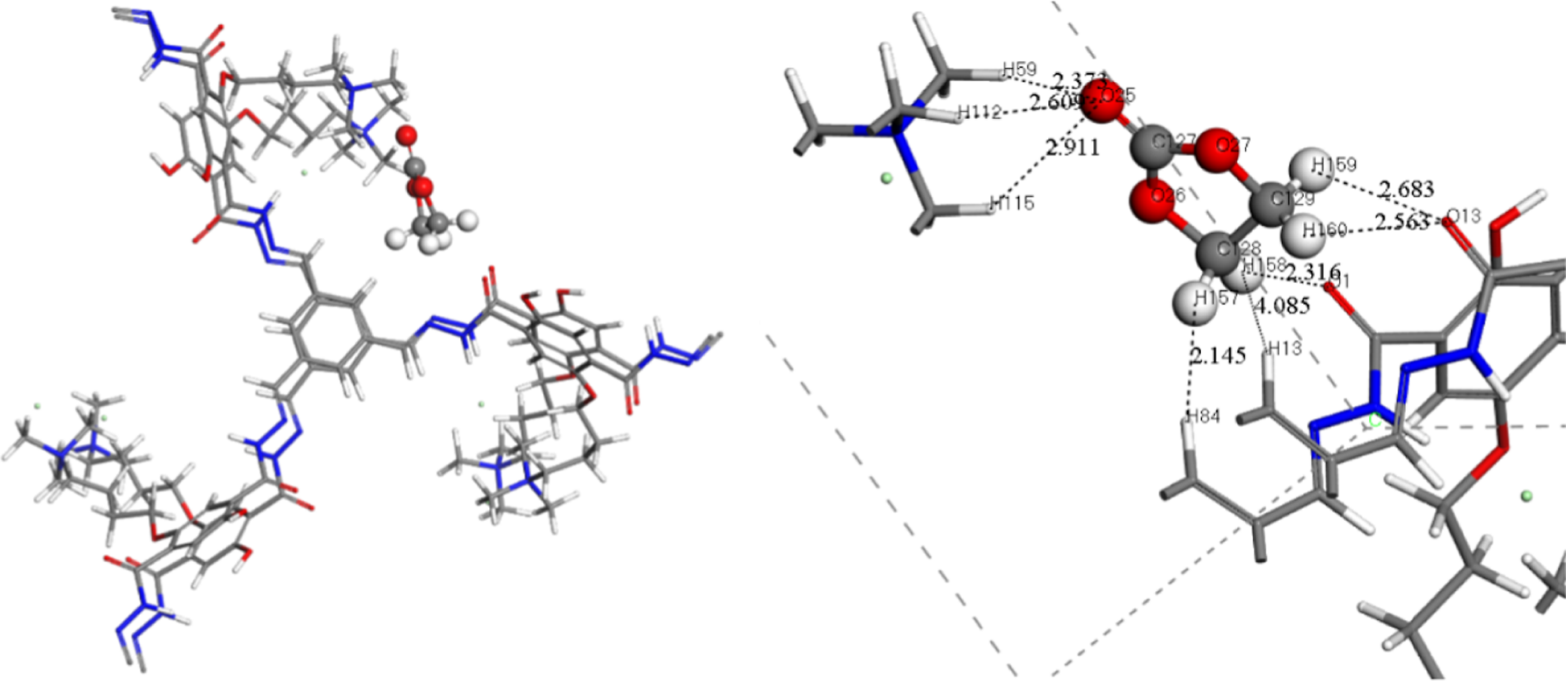
Distance of atoms in EC with COF-QA-4 framework atoms. The white, gray, blue, red, and green balls represent H, C, N, O, and Cl atoms, respectively. (Atoms C127, C128, C129, O25, O26, O27, H157, H158, H159, and H160 belong to the EC molecule; atoms O1, O13, H13, H59, H84, H112, H115 belongs to the COF-QA-4 framework.) Only the framework around the adsorbed EC molecule is shown in this figure.

In order to obtain the most stable configurations of EC molecular adsorption on COF-QA-4, three initial binding configurations (shown in Figure S5) were extracted from a Monte Carlo simulation of a single EC molecule in the COF at 298 K (translation, rotation, and reinsertion moves were attempted). Then, the adsorption energy *E*_adsorption_ of the EC molecule in the COF-QA-4 framework was calculated using DFT calculations after full optimization. All of the values of the adsorption energy of EC on different sites of the COF-QA-4 framework were negative, suggesting that the adsorption of EC in the COF-QA-4 framework was an exothermic process, and EC could interact with COF-QA-4 spontaneously (Table S5). The most stable configuration of the adsorbed EC molecule in COF-QA-4 is depicted in Figure 8, and the adsorption energy *E*_adsorption_ was –120.45 kJ/mol. The strong interactions between the host framework and the guest molecule can be evidenced by the steep and high adsorption at low EC concentration in the adsorption isotherm of trace EC vapor in COF-QA-4.

**Figure 9:**
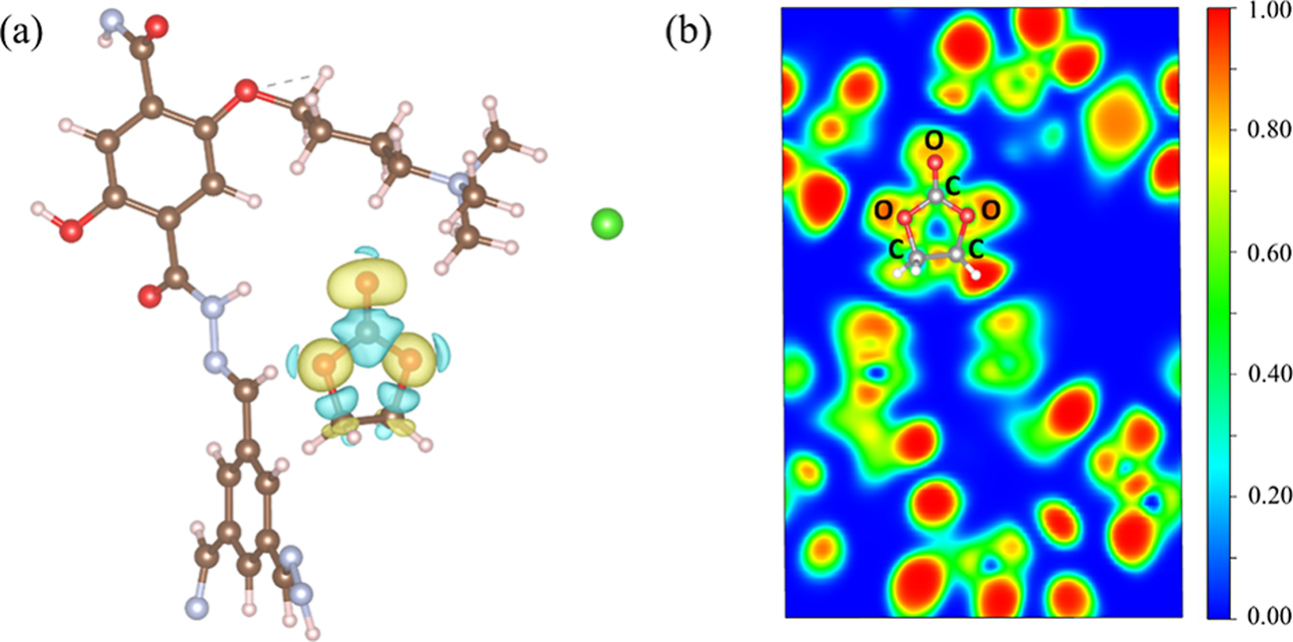
(a) CDD plot for the EC molecule adsorbed in COF-QA-4. The yellow (blue) distribution corresponds to charge accumulation (depletion). The isosurface value is equal to 0.015 e/Å^3^. (b) ELF plot of the EC molecule adsorbed in COF-QA-4. The ELF is projected onto the (110) plane. The blue color area means that the electronic density is zero, and the electronic density gradually increases when the color changes from blue to red.

The charge transfer of the equilibrium adsorption configuration after EC adsorption in the COF-QA-4 framework was also calculated. The amount of charge transfer of the EC molecule was +0.0116 e, which means that the COF-QA-4 framework lost 0.0116 electrons after EC adsorption and served as the electron donor. The CDD plot and ELF plot of EC adsorbed in the COF-QA-4 framework are shown in Figure 9. There were no apparent overlaps of electronic clouds in the area between the EC molecule and the COF-QA-4 framework, revealing no chemical bonding formed after EC adsorption in COF-QA-4.

The DOS and band structures after EC adsorption in the COF-QA-4 framework were calculated for a detailed interpretation of the mechanism. It can be seen that the band gap of COF-QA-4 decreased from 1.515 to 1.366 eV after the EC adsorption, indicating that the conductivity of COF-QA-4 increased after the adsorption of EC (Figure 7b). This is owing to the electron holes increased in COF-QA-4 after the EC adsorption.

**Figure 10:**
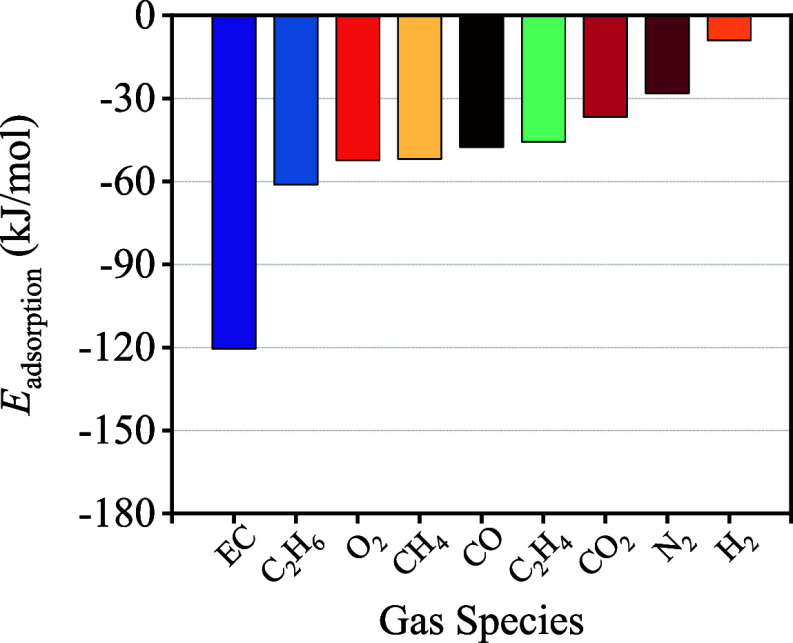
Adsorption energy of EC, N_2_ and different impurity gas in the COF-QA-4 framework calculated by the DFT method.

In order to investigate the selectivity of COF-QA-4, the adsorption energy of different impurity gas molecules and balance gas N_2_ with the COF-QA-4 framework were also calculated and are shown in Figure 10 and Table S8. The EC possessed the highest adsorption energy among all the gases above, which means that COF-QA-4 has the biggest selectivity against other impurity gases. This phenomenon should be verified in future research through real-time sensing performance experiments.

### 3.3 COF Synthesis and Characterization

**Figure 11:**
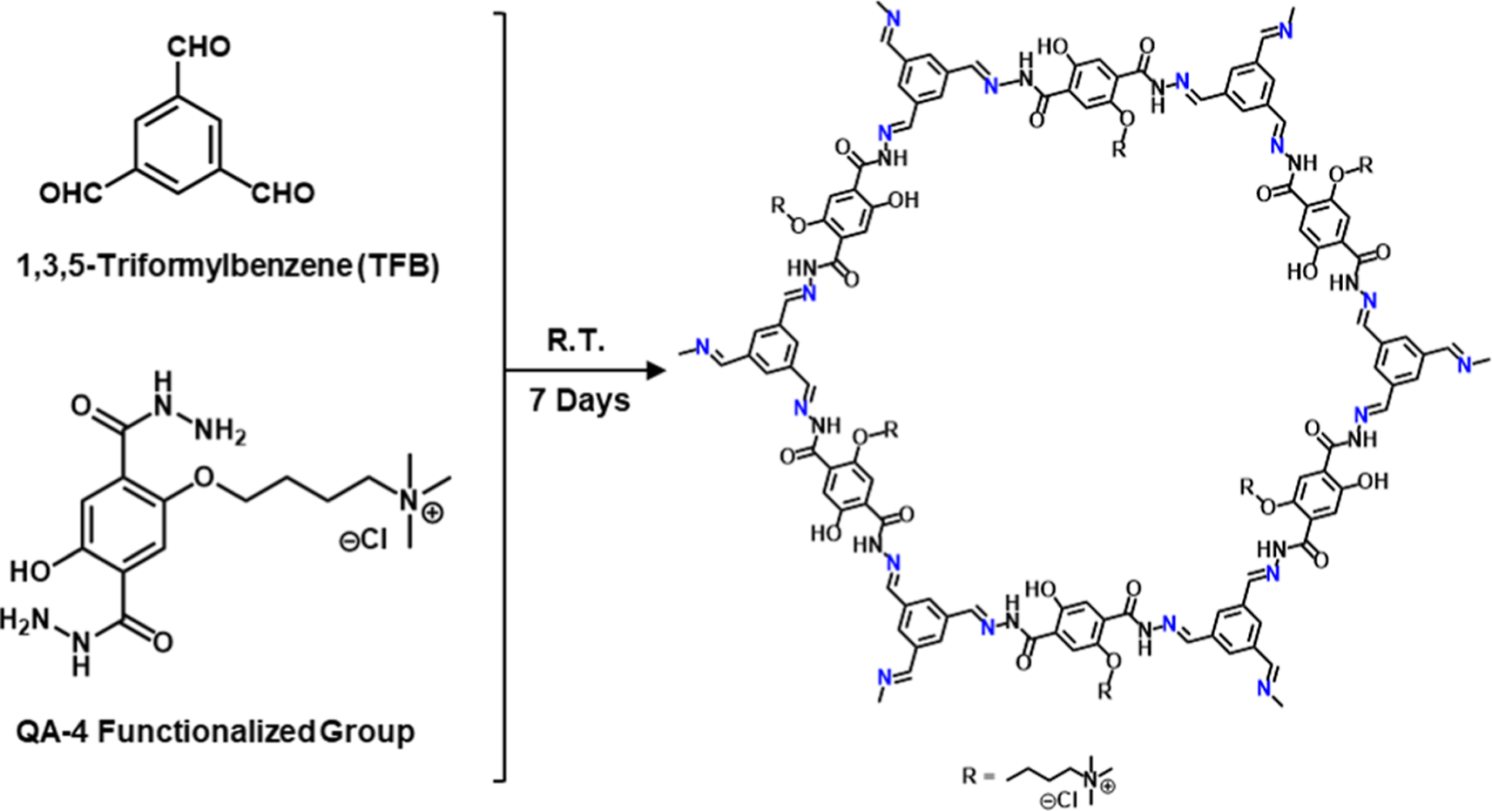
Preparation scheme of COF-QA-4.

Based on the results of GCMC simulations and DFT calculations, COF-QA-4 was identified as a promising candidate and selected for synthesis to evaluate its performance in EC sensing experiments. COF-QA-4 was prepared using phase transfer polymerization through the Schiff-base reaction between TFB and the QA-4 following the reported procedure (Figure 11).^15^ The mesitylene dissolved in TFB was transferred to the upper layer of water containing QA-4 using acetic acid as a catalyst, and then the resultant COF-QA-4 polymerized in the aqueous phase. The aqueous phase was collected to obtain a stable colloidal suspension of COF-QA-4 (Figure S7).

Fourier-transform infrared (FTIR) spectroscopy of COF-QA-4 showed the disappearance of stretching vibrations of H–C=O at 1710–1685 cm^–1^ from TFB, and the appearance of imine bonds (C=N) at 1650–1590 cm^–1^ from the QA-4-functionalized group indicated that COF-QA-4 had been successfully synthesized through the Schiff-base condensation reaction (Figure S8). Elemental analysis showed that C/N/O/Cl ratios were consistent with theoretical values (Table S10), although the content of C was slightly higher than theoretical values, which could be from the pollutants in the air. X-ray photoelectron spectroscopy (XPS) spectra also evidenced the presence of the characteristic binding energy peak of nitrogen in the C=N bond at 399.99 eV (Figure S14).

Powder X-ray diffraction (PXRD) further indicated the formation of the framework. The diffraction peaks observed at around 3.9, 7.5, and 11.9° correspond to the (100), (200), and (220) reflections, respectively. The peak between 23.5° and 25.5° was assigned the (001) plane, corresponding to weak long-range order with layers stacking along the *c* direction. Overall, the PXRD pattern of our synthesized COF-QA-4 is consistent with both the literature data from a previous study^15^ on COF-QA-4 and the simulated curve for the AA stacking mode of COF-QA-4 (Figure S11). Similar broad PXRD patterns of COF materials have been reported by other research groups.^48−50^ The Bragg peak appearing as a diffraction ring in the transmission electron microscopy (TEM) test also implied the random orientation of the COF-QA-4 crystalline domains (Figure S12). Scanning electron microscopy (SEM) revealed the presence of rod-shaped crystallites with lengths ranging from hundreds of nanometers to several micrometers and widths of several nanometers (Figure S13).

### 3.4 Gas Sensing Performance

**Figure 12:**
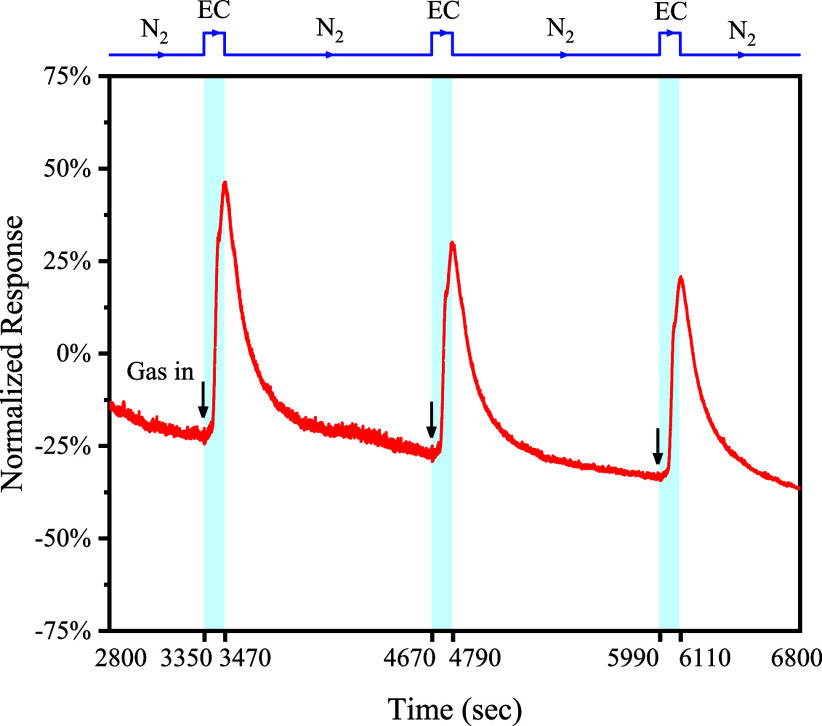
Time-dependent chemiresistive responses of the COF-QA-4-based gas sensor to 1.15 ppmv EC vapor after 120 s exposure under an applied voltage of 1.0 V and an atmosphere of dry nitrogen at room temperature.

The synthesized COF-QA-4 suspension was immobilized on a gold-coated concentric interdigital microelectrode (Figure S16) using the spin-coating method to fabricate the chemiresistive gas sensor (Figure S17). The scheme of the gas sensing system is shown in Figure S20. The amperometry (*I*–*t*) method was implemented by the electrochemical workstation to monitor the current change over time when EC vapor was injected into the testing chamber with balance gas N_2_ under an initial voltage of 1.0 V at room temperature. The analyte gas was injected into the chamber for 120 s, and then the gas sensor was allowed to recover for 20 min in a pure nitrogen environment. The normalized response (*R*) upon the exposure of the EC gas can be expressed by the following formula
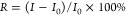
where *I*_0_ is the initial current of baseline, and *I* is the current at various points during measurement after the injection of the EC vapor. The normalized response showed an instantaneous increase trend upon exposure to 1.15 ppmv EC vapor (balance gas N_2_), meaning that the resistance of COF-QA-4 decreased after the EC adsorption. This observation aligns with the DFT calculation results, which indicated an enhancement in the electrical conductivity of COF-QA-4 following the adsorption of EC molecules. Reversibility is a nonnegligible factor of gas sensors that represents reusability under repeated exposures to the analyte. The results showed that the response of COF-QA-4 to the EC was partially reversible after three continuous testing cycles (Figure 12). COF-QA-4 had an average normalized response of 32.46% for three continuous testing cycles, which means this COF-QA-4-based sensor has high sensitivity when exposed to trace EC vapor. As there is no COF-based gas sensor for EC detection, Table S12 gives a summary of the recent advances in gas sensors for OC sensing. Compared with other OC gas sensors, the COF-QA-4-based gas sensor has a good sensing ability.

**Figure 13:**
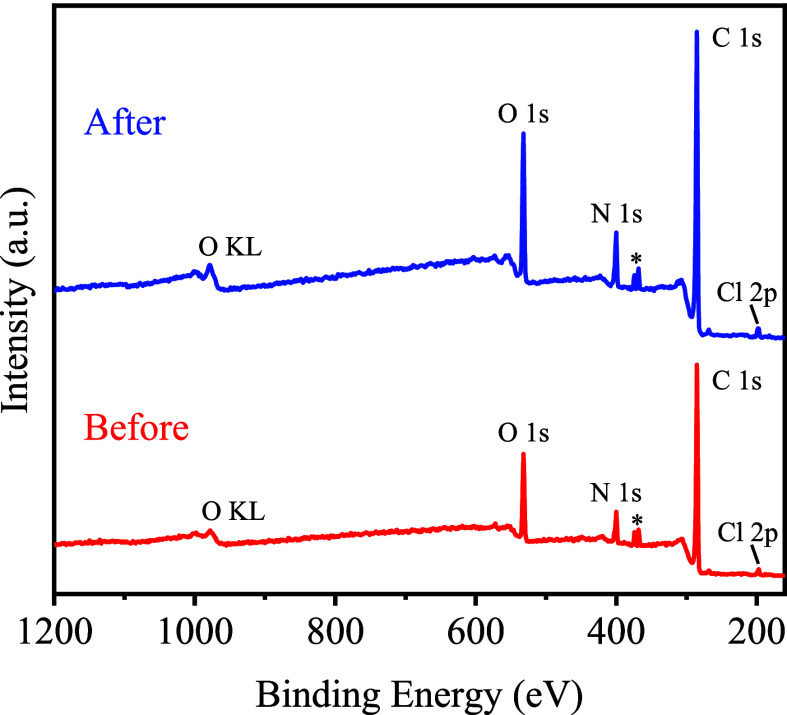
XPS survey spectrum of COF-QA-4 before and after EC adsorption (The peaks at * belonged to the 3d orbitals of residual Ag element).

XPS was performed to investigate the sensing mechanism. Figure 13 shows the surface elemental composition of COF-QA-4 before and after EC adsorption analyzed using a full-range XPS spectrum. Deconvoluted XPS spectra of Cl 2p, C 1s, N 1s, and O 1s orbitals were obtained through narrow-band scanning (Figure S14, Figure S15). The XPS spectrum and deconvoluted XPS spectra of COF-QA-4 exhibited no significant changes in bonding after EC adsorption, confirming the absence of new chemical bond formation. This finding aligns with the ELF analysis from DFT calculations. Both experimental results and theoretical calculations suggest that the interactions between EC molecules and the COF-QA-4 framework are primarily governed by reversible interactions and charge transfer processes under the tested conditions.

## 4 Conclusions

In this study, we employed a computationally guided approach to design COF-based chemiresistive gas sensors for the selective detection of trace EC vapor and elucidated its sensing mechanism. We have demonstrated that high-throughput GCMC simulations complemented by DFT calculations were promising in identifying promising COF candidates from the CURATED COF database for the selective and efficient capture of trace EC vapor. The simulation results showed that COFs selected with PLD of 5.98–21.34 Å, ASA of 1149.92–4633.23 m^2^/g, HVF of 0.461–0.738, and *Q*^0^_st_ of –111.64 ∼ –70.03 kJ/mol exhibited strong potential for selective adsorption of trace EC vapor. Among these candidates, COF-QA-4 emerged as the most promising material due to its superior selectivity toward EC compared with other gas impurities. The use of HTS with GCMC simulations proved to be a cost-effective and efficient strategy for narrowing down the candidate pool, providing a significant advantage over relying solely on DFT calculations (Table S9). DFT calculations were subsequently employed to validate the GCMC results and further investigate the sensing mechanism, revealing that COF-QA-4 interacts with EC molecules through a charge transfer mechanism. The adsorption energy of EC on COF-QA-4 was calculated to be –120.45 kJ/mol, and the results showed that EC adsorption leads to a reduction in the band gap of COF-QA-4, consistent with the observed decrease in the resistance during gas sensing tests.

Following the computational screening, experimental efforts focused on the synthesis of COF-QA-4 through phase transfer polymerization, using a Schiff-base reaction between TFB and QA-4. The synthesized COF-QA-4 was then incorporated into chemiresistive gas sensors. These sensors exhibited a rapid and detectable response to 1.15 ppmv EC vapor within 120 s, with stable reversibility over three consecutive testing cycles.

This research represents the first reported development of a COF-based chemiresistive sensor for the selective detection of EC vapor, demonstrating the value of integrating computational screening with experimental validation. Our findings have significant implications for the design of porous materials for real-time electrolyte leakage detection in LIBs under ambient conditions. Moving forward, future studies will focus on optimizing the practical deployment of COF-QA-4-based sensors in operational environments. Additionally, this work highlights a novel strategy for leveraging computational methods to screen and develop advanced materials for targeted sensing applications. The methodology outlined here offers a blueprint for future research in designing high-performance sensing materials that enables faster and more efficient development cycles.

## Supporting Information Available

Instrument and chemicals; the establishment of the simulation model; GCMC simulations; DFT study; synthesis procedure; COF characterization; the gas-sensing platform; and Characterization of monomer (PDF)
