# Supplementary material for: Computational Screening Guiding the Development of a Covalent-Organic Framework-Based Gas Sensor for Early Detection of Lithium-Ion Battery Electrolyte Leakage
Source: ACS Appl Mater Interfaces. 2025 Feb 3;17(6):10108–17. doi: 10.1021/acsami.4c19321 (PMC11826885; doi:10.1021/acsami.4c19321)
Supplement: Supplementary file 1 — am4c19321_si_001.pdf [file am4c19321_si_001.pdf]

Supporting Information for

Computational Screening Guiding the Development of a Covalent-Organic Framework-Based Gas Sensor for Early Detection of Lithium-Ion Battery Electrolyte Leakage

Liangdan Zhao[a,b], Chunyi Yu[a,b], Xiaoyu Wu[c], Mingrui Zuo [a,b], Qian Zhang[a], Qiuchen Dong[a], and Lifeng Ding*[a,b]*

[a] *Department of Chemistry and Advanced Materials Research Center, School of Science, Xi'an Jiaotong-Liverpool University, Suzhou, Jiangsu Province, 215123, P. R. China*

[b] *Department of Chemistry and Materials Innovation Factory, University of Liverpool, Liverpool, L69 7ZD, United Kingdom*

[c] *Department of Chemical and Biomolecular Engineering, National University of Singapore, 117585, Singapore*

Emails: Qiuchen.Dong@xjtlu.edu.cn (Q.D.), Lifeng.Ding@xjtlu.edu.cn (L.D.)

Contents	Page No.
1. Instrument and Chemicals	3
2. The establishment of the simulation model	5
3. GCMC simulation	9
4. DFT study	11
5. Synthesis procedure	13
6. COF characterization	16
7. The gas-sensing platform	21
8. Characterization of monomer	26
9. Reference	34

1. Instrument and Chemicals

1.1 Instrument

Nuclear magnetic resonance (NMR) spectroscopy:

The ^{1}H and ^{13}C NMR spectra of the monomer were characterized by Bruker Ascend 400 NMR spectrometer (400 MHz for ^{1}H NMR, 100 MHz for ^{13}C NMR).

Fourier-transform infrared spectroscopy (FTIR):

Agilent Cary 630 FTIR spectrometer was used for FTIR measurements with signal averaging values of 64 scans at 4 cm^{-1} resolution at room temperature.

Powder X-ray diffractometry (XRD):

Powder X-ray diffractometry (PXRD) patterns were carried out on a Bruker D8 Advance diffractometer with a Cu Kα source (λ = 0.1541 nm) to examine the crystallinity of the COF. The data was collected within the 2θ range of 2°-40°.

Thermogravimetric analysis (TGA):

The NETZSCH STA 449 F5 Jupiter instrument was employed to conduct thermogravimetric analysis. The sample was analyzed at 298K under the N_2 atmosphere with a flow rate range from 25 mL/min to 100 mL/min.

Scanning Electron Microscope (SEM):

JEOL JSM 6510 was employed to obtain the high-magnification images of materials under 5 kV working voltage.

Water contact angle measurements:

The experiments were conducted using a Biolin Scientific Attension Theta Lite optical tensiometer at 25 ± 2 °C.

X-ray photoelectron spectroscopy (XPS):

XPS was conducted on Thermo Scientific ESCALAB 250Xi equipment.

Transmission electron microscope (TEM):

The transmission electron microscope (TEM) was carried out at an FEI G2 F30 TEM instrument under 300 kV working voltage.

Mass spectrometry:

The mass spectra were characterized by quadrupole time-of-flight liquid chromatography-mass spectrometry (Q-TOF-LC-MS) using Agilent 1260 Infinity HPLC system and Bruker MicrOTOF-Q II instrument.

1.2 Chemicals

Most of the reagents were purchased from Shanghai Bide Pharmaceutical Technology Co., Ltd., including 2,5-dihydroxyterephthalic acid diethyl ester (99.57%), potassium carbonate (99.03%), potassium iodide (99.1%), 1,4-dibromobutane (99.94%), silver chloride (99.56%) and 1,3,5-triformylbenzene (96%). Tetrabutylammonium iodide (⩾98%) was purchased from Accela ChemBio Co., Ltd. Mesitylene (99%), N,N-dimethylformamide (99.8%) and 1,4-dioxane (99.7%) were purchased from Adamas-beta. Trimethylamine (33%) solution in ethanol was purchased from Thermo Fisher Scientific Inc. Acetone, ethanol, ethyl acetate and n-hexane were purchased from Shanghai Lingfeng Chemical Reagent Co., Ltd. Acetic acid was purchased from General-Reagent. Hydrazine hydrate (85%) was purchased from Sinopharm Chemical Reagent Co., Ltd. Ethylene carbonate (99%) was bought from Shanghai Aladdin Bio-Chem Technology Co., Ltd.

2. The establishment of the simulation model

Table S1. Calculated characteristics of isolated EC molecule compared with the previous calculations and experiments.

	This work	Previous work[1]	
Geometries	B3LYP/6-31G(d,p)	Calculated	Experiments
Bond length (Å)			
O1-C2	1.194	1.193	1.203
C2-O3/C2-O5	1.364	1.360	1.342
O3-C4/O5-C6	1.434	1.430	1.457
C4-C6	1.534	1.520	1.522
C4-H8/C4-H10	1.091	1.080	1.091
C4-H7/C6-H9	1.095	1.080	1.091
Bond angle (°)			
O1-C2-O3	124.890	124.95	124.17
C2-O3-C4	109.456	109.43	108.71
O3-C2-O5	110.220	110.10	111.67
O5-C6-H10	108.934	108.78	108.30
C6-C4-H7	112.558	112.55	113.94
H9-C6-H10	109.487	109.81	110.82

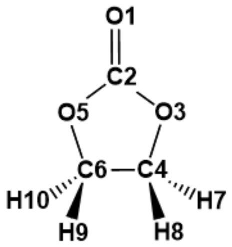

The cylindrical model was considered to describe the minimum molecular dimension for ethylene carbonate (EC).[2] The diameter of the minimal enclosing cylinder (circumscribed cylinder) of the ethylene carbonate (EC) molecule (D_{cyl}) was calculated using the CYL script.[3] The diameter of the minimal enclosing cylinder plus twice the van der Waals radius of the oxygen atom served as the minimum molecular dimension (D_{min}), which can exactly enclose the EC molecule. Here, the D_{min} was calculated as 5.9 Å (**Figure S1**).

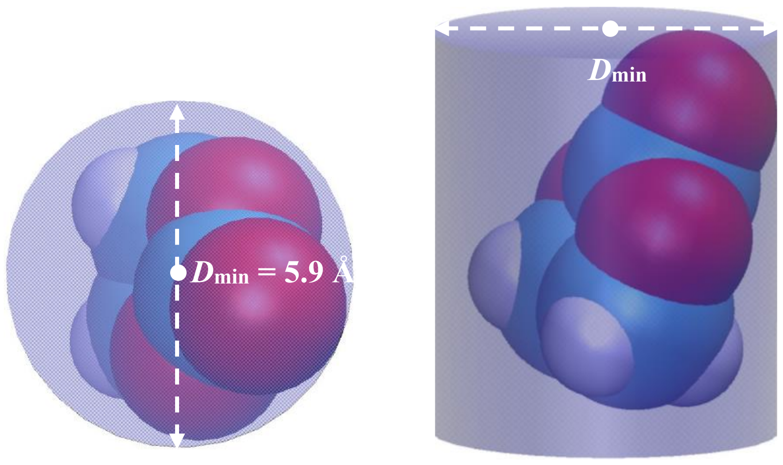

Figure S1. The scheme for the minimum molecular dimension (D_{min}) of the EC molecule.

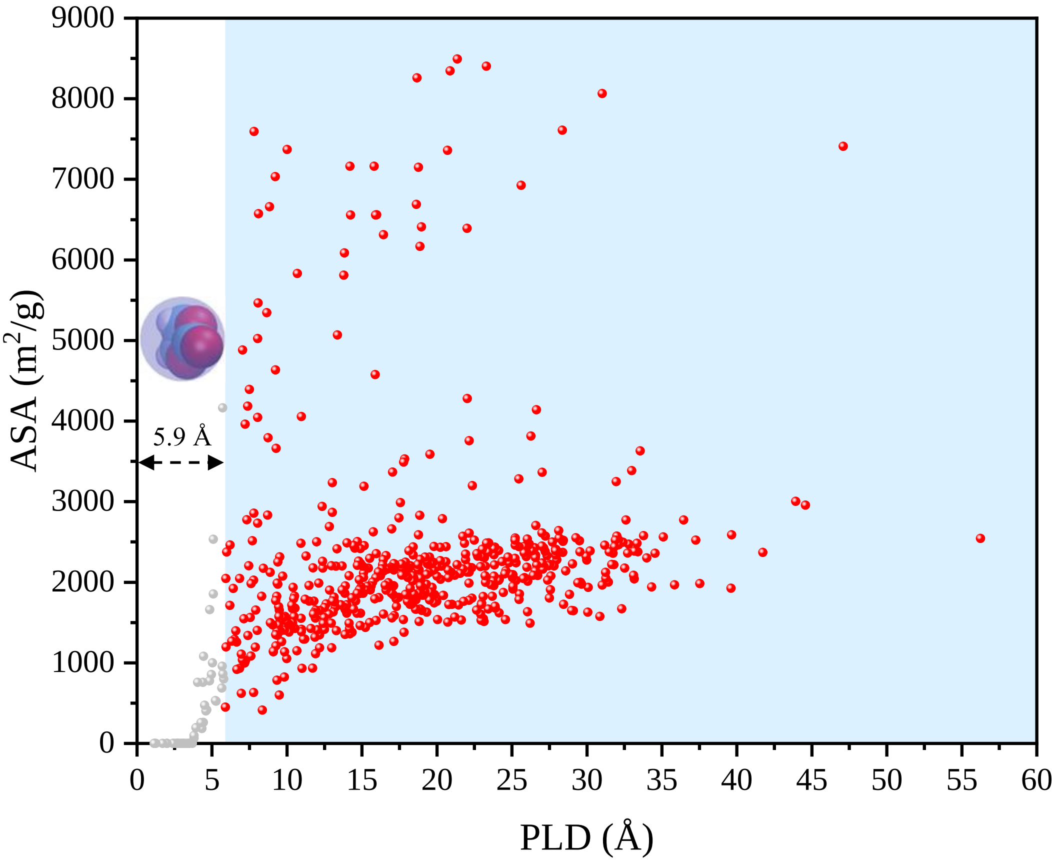

Figure S2. The calculated PLD and ASA data of 612 COFs in the database. Grey data points: COFs with PLD ≤ 5.9 Å, ASA = 0 m^2/g; Red data points: COFs with PLD > 5.9 Å, ASA > 0 m^2/g.

Table S2 and Table S3 listed the force field parameters and partial charges used in the intermolecular potentials for nonbonded interactions of EC molecules.

Table S2. OPLS-AA force field parameters and partial charges used in the intermolecular potentials for nonbonded interactions of EC molecule[4-6]

Atom	σ (Å)	ϵ/k_B (K)	q (e)
O1	2.96	105.678	-0.51986
C2	3.75	52.839	0.835588
O3	3.00	85.549	-0.39489
C4	3.50	33.213	0.145592
O5	3.00	85.549	-0.39489
C6	3.50	33.213	0.145592
H7	2.50	15.097	0.04421
H8			0.047226
H9			0.04421
H10			0.047226

Table S3. TraPPE-AA force field parameters and partial charges used in the intermolecular potentials for nonbonded interactions of EC molecule[7-8]

Atom	σ (Å)	ϵ/k_B (K)	q (e)
O1	3.05	79	-0.51986
C2	3.82	40	0.835588
O3	2.80	55	-0.39489
C4	3.95	46	0.145592
O5	2.80	55	-0.39489
C6	3.95	46	0.145592
H7	2.36	25.45	0.04421
H8			0.047226
H9			0.04421
H10			0.047226

Table S4. Force field parameters used in the intermolecular potentials for nonbonded interactions of impurity gas molecules

Molecule	Atom	ε/k_B (K)	σ (Å)	Reference
CO	C	52.888	3.43	Ref.9
	O	30.219	3.12	
N_2	N	36.0	3.31	Ref.10
	N_com	0.0	0.0	
CO_2	C	27.0	2.80	Ref.10
	O	79.0	3.05	
H_2O	H_spcE	0.0	0.0	Ref.11
	O_spcE	78.20	3.1656	
O_2	O	49.0	3.02	Ref.12
	O_com	0.0	0.0	
H_2	H	0.0	0.0	Ref.13
	H_com	36.7	2.96	
CH_4	CH_4	148.0	3.73	Ref.14
C_2H_4	CH2_sp2	85.0	3.675	Ref.15
C_2H_6	CH3_sp3	98.0	3.75	Ref.14

3. GCMC simulation

3.1 Henry's adsorption constant (K_H) and isosteric heat of adsorption at zero coverage (Q^0_{st})

Henry's adsorption constant (K_H) is the constant appearing in the linear adsorption isotherm, which represents a weak binding ability of the framework with guest molecules. Henry's adsorption isotherm is the simplest adsorption isotherm in that the amount of the surface adsorbate is represented to be proportional to the partial pressure of the adsorptive gas:[16]

$$X = K_H P \quad \text{...S(1)}$$

where X is surface coverage, P is partial pressure, and K_H is Henry's adsorption constant. The linear isotherm can describe the initial part of many practical isotherms and is typically considered valid for low surface coverages.

The enthalpy of adsorption at infinite dilution (also known as isosteric heat of adsorption at zero coverage, Q^0_{st}) is equal to $\langle U_{hg} \rangle - RT$.

When gas is on the homogeneous adsorbent in the very low pressure (Henry's law) region, the isosteric heat of adsorption remains constant at low adsorbate loadings:

$$lnK_H = -\frac{Q^0_{st}}{RT} + \mathrm{C} \quad \text{...S(2)}$$

where K_H is Henry's adsorption constant, Q^0_{st} is isosteric heat of adsorption at zero coverage.

3.2 Selectivity of impurities and adsorption isotherm of 12 COFs

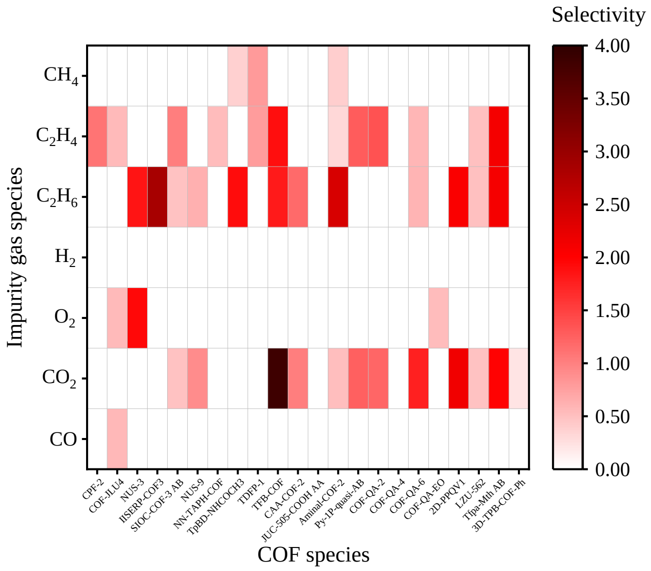

Figure S3. Selectivity of 1 ppmv impurities CO, CO_2, O_2, H_2, CH_4, C_2H_4 and C_3H_6 against 1 ppmv EC gas.

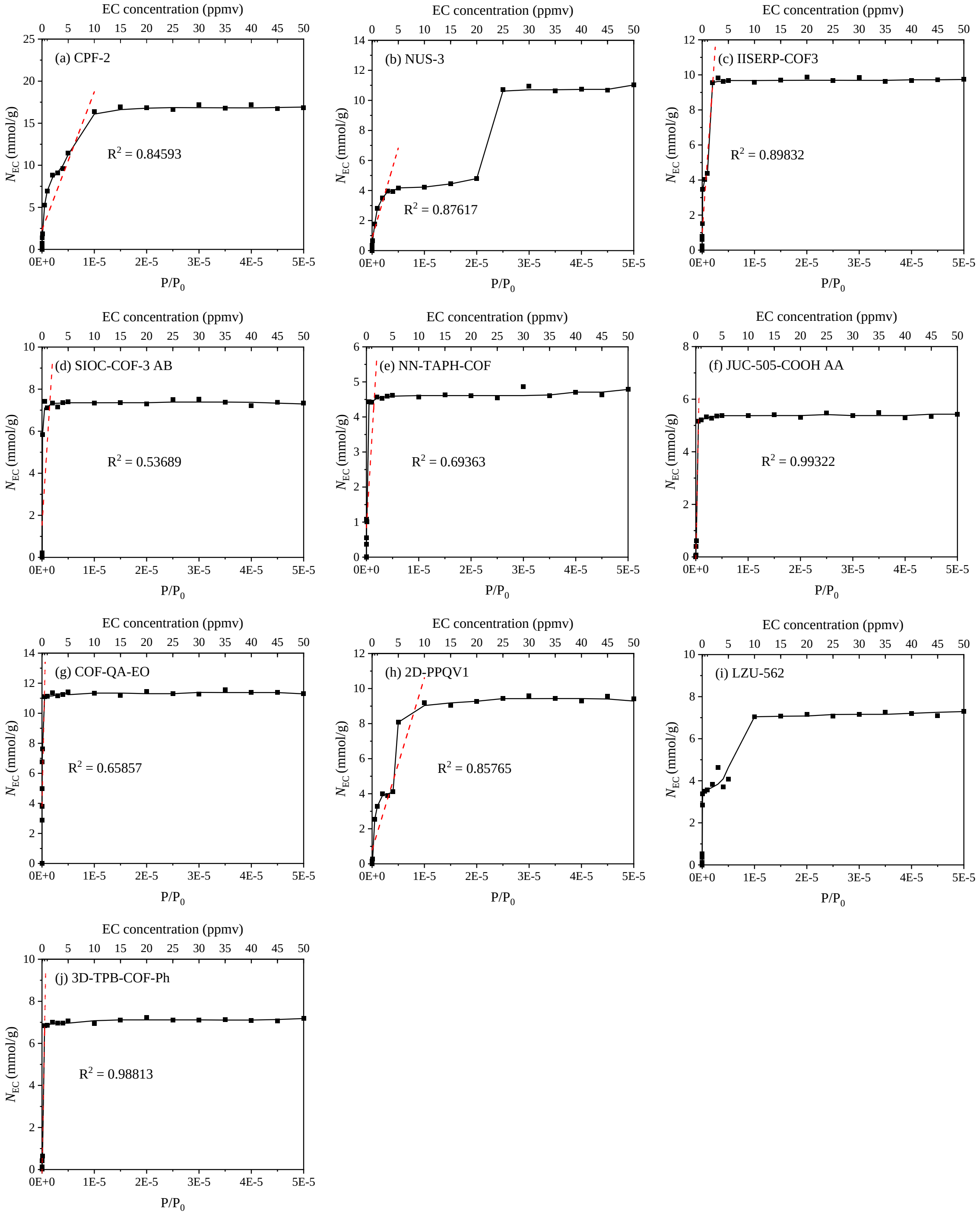

Figure S4. EC adsorption isotherms of (a) CPF-2 (b) NUS-3 (c) IISERP-COF3 (d) SIOC-COF-3 AB (e) NN-TAPH-COF (f) JUC-505-COOH AA (g) COF-QA-EO (h) 2D-PPQV1 (i) LZU-562 and (j) 3D-TPB-COF-Ph under 1 bar pressure with N_2 at 298 K from GCMC simulations.

4. DFT study

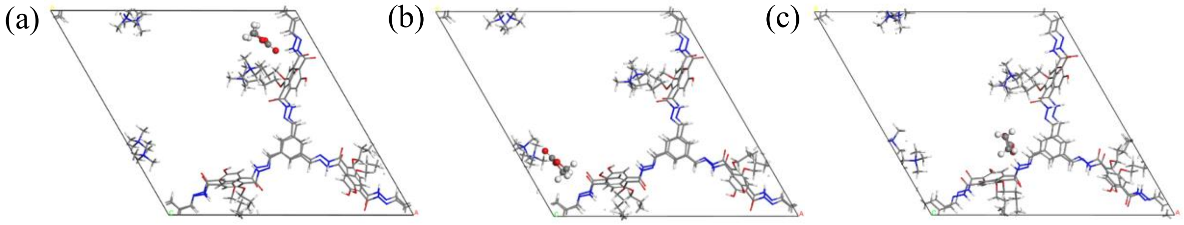

Figure S5. Optimized host-guest interaction configurations of adsorbed EC molecule in COF-QA-4 framework at site 1 (a), site 2 (b) and site 3 (c).

Table S5. The adsorption energy of EC molecule with COF-QA-4 at different adsorption sites

Adsorption configuration	Site 1	Site 2	Site 3
$E_{adsorption}$ (kJ/mol)	-43.46	-120.45	-21.87

The Q^0_{st} calculated using different combinations of force field parameters by GCMC simulations and binding energy calculated using the DFT method by VASP were compared to verify the viability of the force field used in GCMC calculations. **Table S6** showed that the UFF for the COF framework and OPLS force field for the EC molecule were the preferred choices in GCMC simulations. The Q^0_{st} calculated by GCMC simulation and adsorption energy calculated using the DFT method are in agreement for both cases. Therefore, these results support the idea that GCMC simulations using the UFF and OPLS force fields would be suitable for describing EC vapour captured by COFs.

Table S6. Q^0_{st} of the EC molecule in COF-QA-4 calculated using different force fields

Force Fields	Q^0_{st} (kJ/mol)
UFF-OPLS	-104.41
UFF-TraPPE	-100.88
Dreiding-OPLS	-95.40
Dreiding-TraPPE	-96.06

Table S7. Valence electron charges and charge transfer (in e) of EC molecule determined by Bader charge analysis

	Valence charge		Charge transfer	
Atom	Before	After	Before	After
C127	2.788128	2.246737	-1.211872	-1.753263
C128	3.727939	3.613391	-0.272061	-0.386609
C129	3.69498	3.566879	-0.30502	-0.433121
O25	5.716788	7.093728	-0.283212	1.093728
O26	5.597502	6.818281	-0.402498	0.818281
O27	5.591013	6.831949	-0.408987	0.831949
H157	0.955085	0.998453	-0.044915	-0.001547
H158	0.973657	0.905138	-0.026343	-0.094862
H159	0.987319	0.992762	-0.012681	-0.007238
H160	0.967589	0.944247	-0.032411	-0.055753

Table S8. The adsorption energy $E_{adsorption}$ of different gas molecules with COF-QA-4

Gas species	$E_{adsorption}$ (kJ/mol)
EC	-120.45
C_2H_6	-61.15
O_2	-52.33
CH_4	-51.85
CO	-47.59
C_2H_4	-45.70
CO_2	-36.68
N_2	-28.14
H_2	-9.02

Table S9. The comparison of CPU hours consumption for different calculation methods

Calculation method	Average consumption for 1 COF (CPU hours)
HTS using GCMC simulation	167.3
DFT calculation	12,584.6

5. Synthesis procedure

5.1 Synthesis of *2-hydroxy-5-(trimethylammonio-butoxy)-terephthalohydrazide* (QA-4 functionalized group)

2-hydroxy-5-(trimethylammonio-butoxy)-terephthalohydrazide (hereinafter referred to as **QA-4** functionalized group) were synthesized following X. He et al.'s method (**Figure S6**).[17] The products of each step were characterized by ^{1}H NMR and ^{13}C NMR (**Figure S22-Figure S27**).

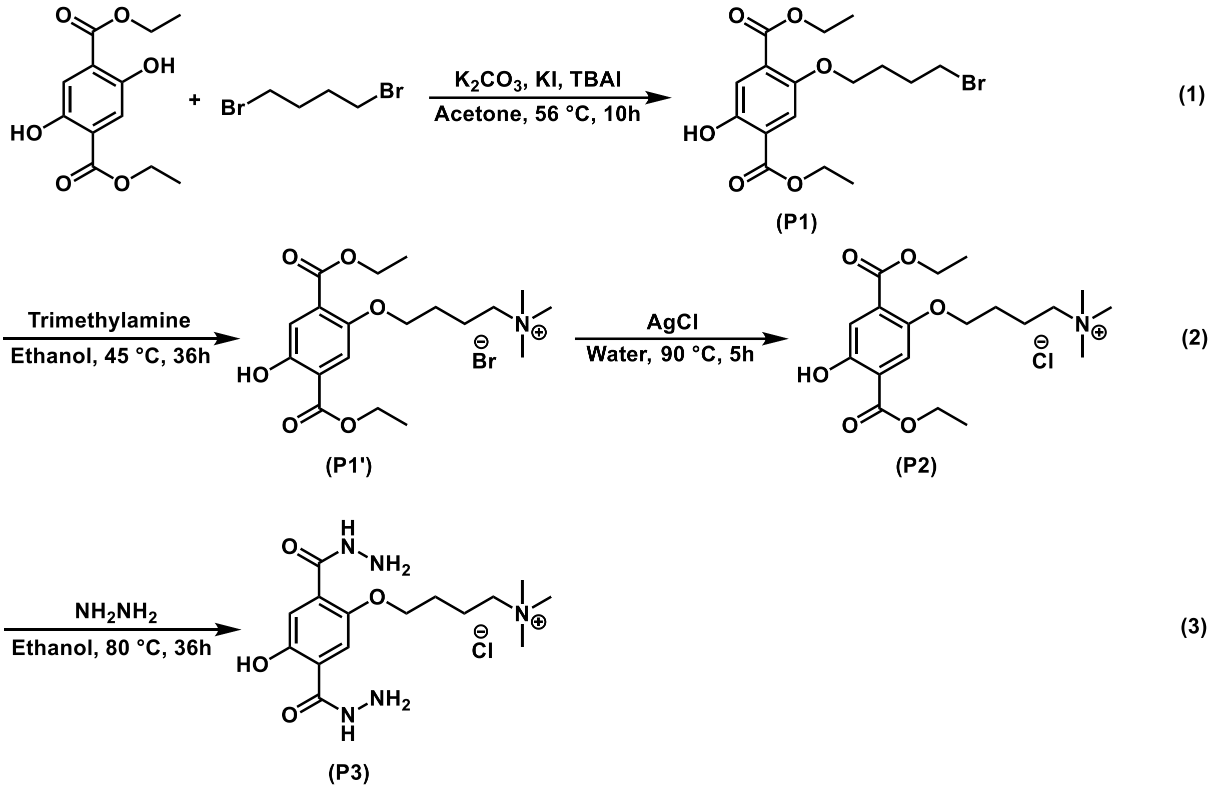

Figure S6. Synthesis procedure of the QA-4 functionalized group.

(1) Synthesis of *2-hydroxy-5-(4-bromobutoxy)-terephthalic acid diethyl ester* (**P1**)

2,5-dihydroxyterephthalic acid diethyl ester (3.05 g, 12.0 mmol) was dissolved in 250 mL of acetone, with potassium carbonate (8.29 g), potassium iodide (450 mg) and Tetra-n-butylammonium iodide (36.9 mg, 0.1 mmol) was added. Then, 1,4-dibromobutane (10.36 g, 48.0 mmol) was added to the mixture and heated to 56 °C for 10 h under nitrogen protection. The filtrate was evaporated to obtain the crude

product. The crude product was purified using a chromatographic column (mobile phase n-hexane/ethyl acetate: 20/1) to get **P1** (1.79 g, 4.6 mmol).

(2) Synthesis of *2-hydroxy-5-(trimethylammonio-butoxy)-terephthalic acid diethyl ester* (**P2**)

P1 (1.79 g, 4.6 mmol) was dissolved in 30 mL of ethanol, adding 5 mL of 33% trimethylamine solution. The mixture was heated to 45 °C for 36 h under nitrogen protection and filtered to get product **P1'**. Afterwards, **P1'** was dissolved in 20 mL of water with silver chloride (1.31 g, 9.2 mmol). Filtrated and evaporated to get product **P2** (1.62 g, 93% yield) after heating at 90 °C for 5 h under nitrogen protection.

(3) Synthesis of *2-hydroxy-5-(trimethylammonio-butoxy)-terephthalohydrazide* (**P3**)

P2 (1.62 g, 4.3 mmol) was dissolved in 50 mL of ethanol with an excessive dose of 85% hydrazine hydrate. The mixture reacted at 80 °C for 36 h and evaporated to get the crude **P3**. The crude product was washed using ethanol and vacuum-dried at 45 °C for 12 h to get **QA-4** functionalized group **P3** (1.49 g, 92% yield).

5.2 Synthesis of the COF-QA-4 membrane

The COF-QA-4 membrane was prepared by phase-transfer polymerization in an organic-aqueous two-phase system. **QA-4** functionalized groups (0.10 mmol) were dissolved in 19 mL of water and 1 mL of acetic acid as the catalyst to get the aqueous phase. 1,3,5-triformylbenzene (**TFB**, 0.07mmol, 10.8 mg) was dissolved in 20 mL of mesitylene to get the organic phase. The organic phase was then transferred onto the top of the aqueous phase for a 7-day reaction at room temperature. Due to the hydrophilicity of ionized hydrazine and the protonation of amino groups, the COF-QA-4 colloidal suspension will finally form in the aqueous phase of the liquid-liquid two-phase system.[18] The schematic of the reaction process within the organic-aqueous system is illustrated in **Figure S7**.

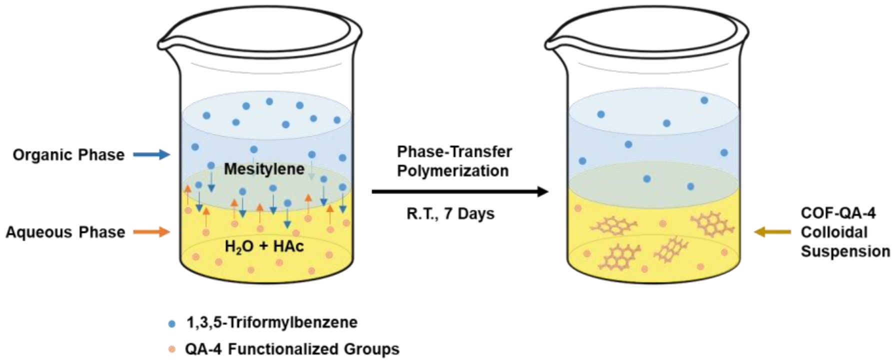

Figure S7. Schematic of the phase-transfer polymerization reaction process of COF-QA-4.

6. COF characterization

The COF-QA-4 colloidal suspension prepared in **Section 5.2** was collected and dried at 60 °C for 24 hours, then washed with water, *N, N*-dimethylformamide, ethanol, and acetone to remove impurities. Finally, the resultant COF-QA-4 membrane was obtained for the following characterizations.

6.1 Fourier-transform infrared (FTIR) spectroscopy

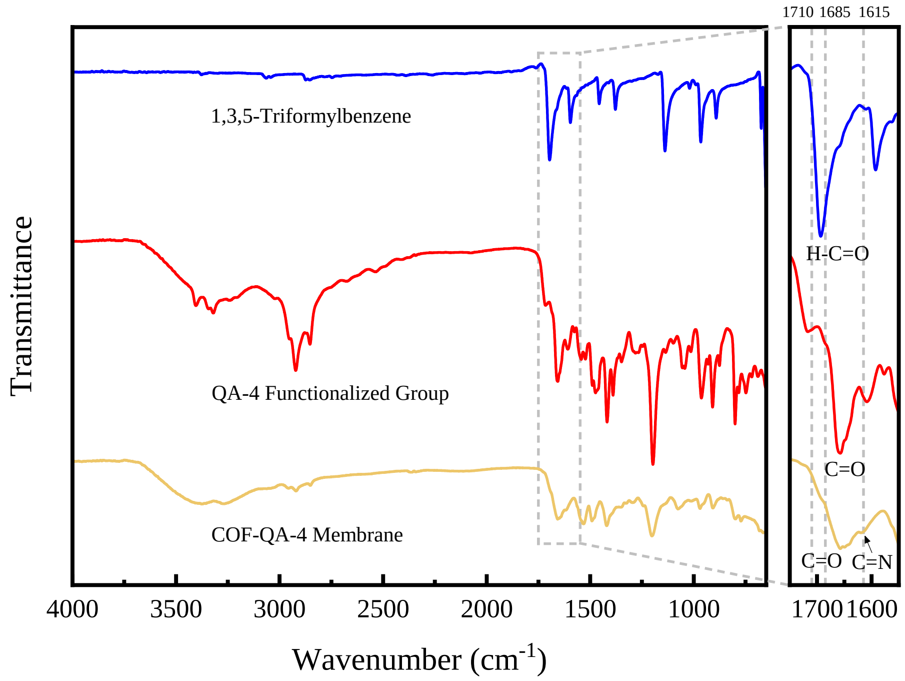

Figure S8. FTIR spectra of COF-QA-4.

6.2 Energy-dispersive X-ray spectroscopy (EDX)

Table S10. Elemental analysis of the COF-QA-4 membrane obtained from theoretical calculation and XPS survey

Element (wt.%)	COF-QA-4 ($C_{126}H_{156}N_{30}O_{24}Cl_6$)	
	The.	Exp.
C	56.31	61.31
N	15.64	16.30
O	14.30	17.34
H	5.81	-
Cl	7.91	5.06

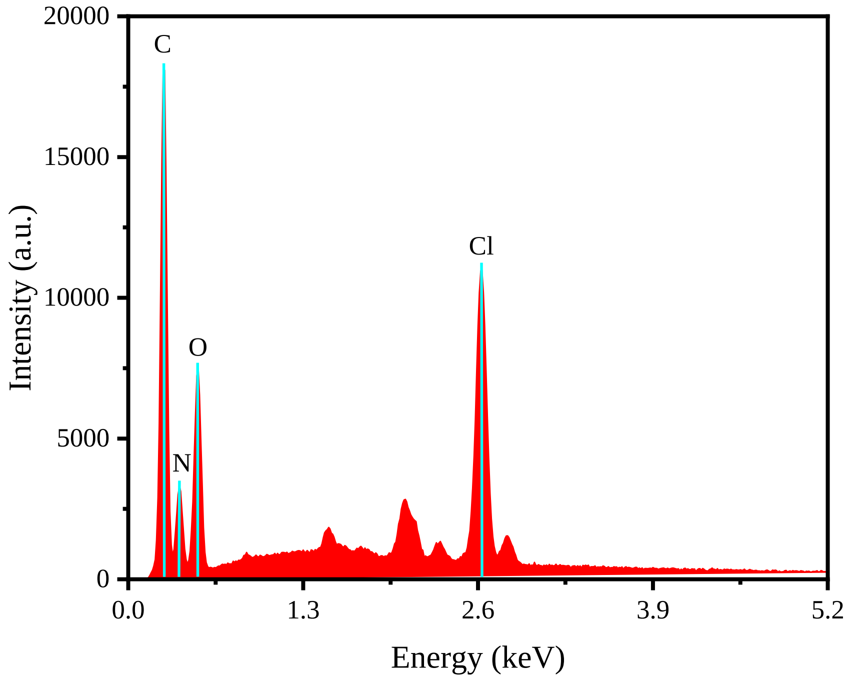

Figure S9. The EDX spectrum of COF-QA-4.

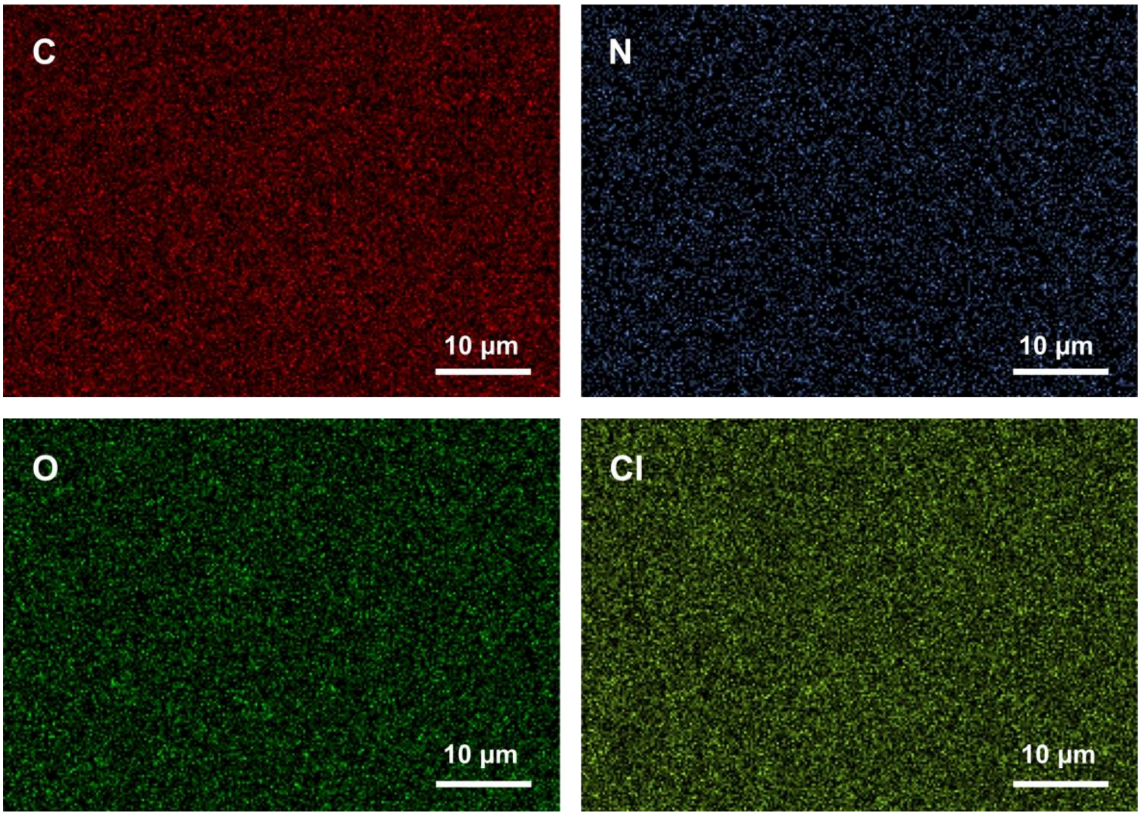

Figure S10. The EDX mapping images of C, N, O, and Cl elements in COF-QA-4.

6.3 Powder X-ray Diffraction (PXRD) Spectra

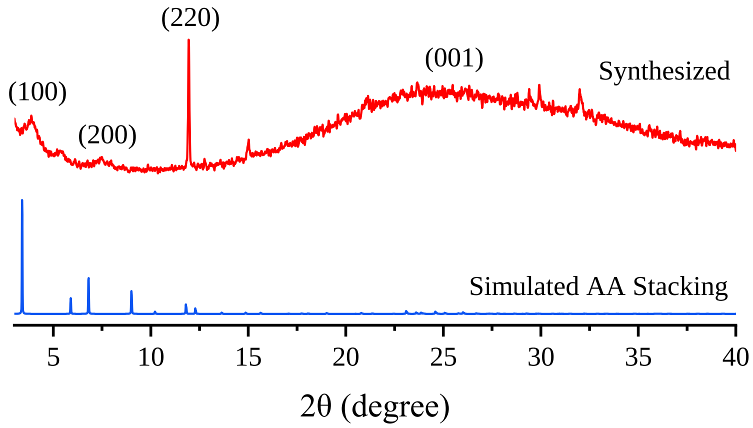

Figure S11. PXRD patterns of the experimental synthesized (red curve) and simulated AA stacking mode (blue curve) of COF-QA-4.

6.4 Transmission electron microscope (TEM)

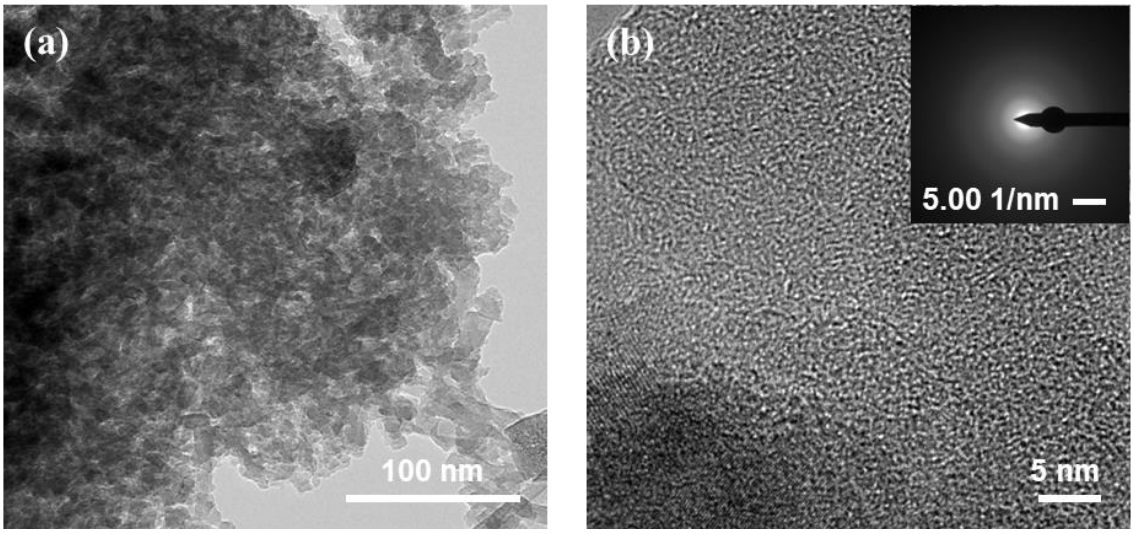

Figure S12. TEM images of COF-QA-4 (a) and its corresponding magnified images (b).

6.5 Scanning Electron Microscope (SEM)

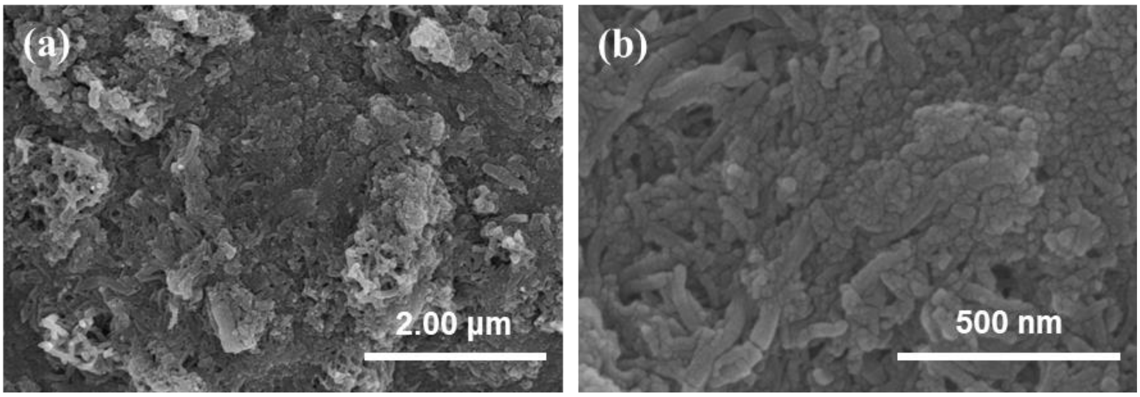

Figure S13. SEM images of COF-QA-4 at (a) low and (b) high magnifications.

6.6 X-ray photoelectron spectroscopy (XPS) spectra

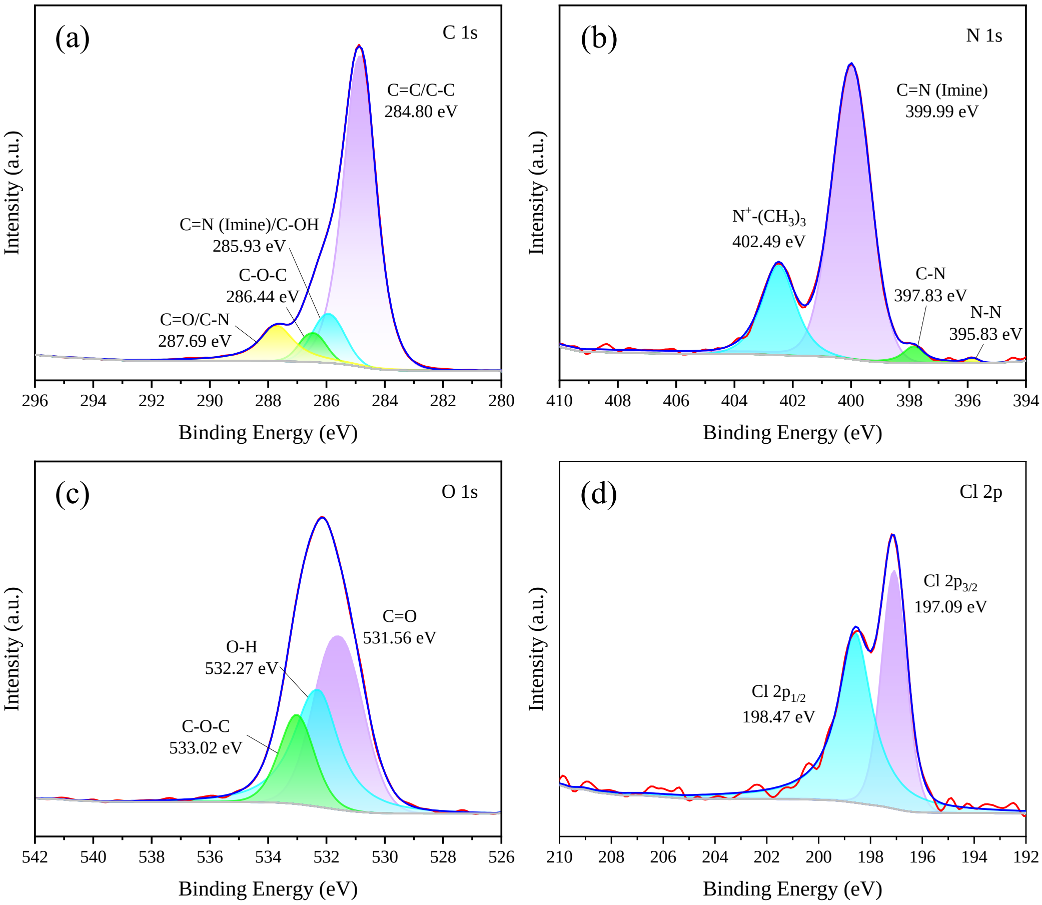

Figure S14. Deconvoluted XPS spectra of COF-QA-4 (a) C 1s, (b) N 1s, (c) O 1s, and (d) Cl 2p.

In **Figure S14a**, the binding energy peaks located at 284.80, 285.93, 286.44 and 287.69 eV were attributed to C=C/C-C, C=N/C-OH, C-O-C and C=O/C-N of C 1s, respectively.[19-21] The peaks spectrum

for N 1s (**Figure S14b**) were attributed to N-N (395.83 eV), C-N (397.83 eV), C=N (399.9 eV), and N^+-$(CH_3)_3$ (402.49 eV), respectively.[22-25] For the O 1s spectra (**Figure S14c**), three peaks are observed, which were assigned to the C=O (531.56 eV), O-H (532.27 eV) and C-O-C (533.02 eV) groups.[26] As displayed in **Figure S14d**, Cl 2p peaks occurred as doublets of Cl $2p_{1/2}$ and Cl $2p_{3/2}$ peaks with binding energies of 197.09 eV and 198.47 eV.[27] The XPS results indicated the successful synthesis of COF-QA-4.

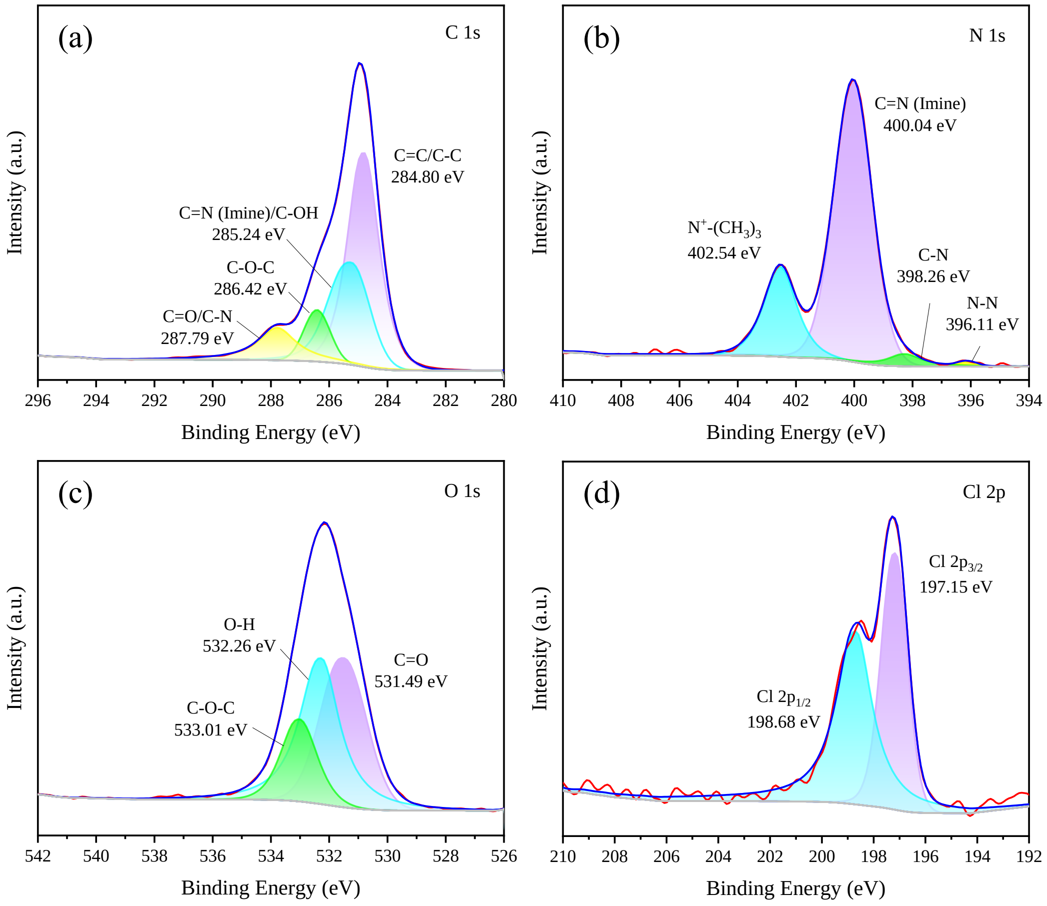

Figure S15. Deconvoluted XPS spectra of COF-QA-4 after EC adsorption: (a) C 1s, (b) N 1s, (c) O 1s, and (d) Cl 2p.

7. The gas-sensing platform

7.1 Fabrication of chemo-resistive gas sensors

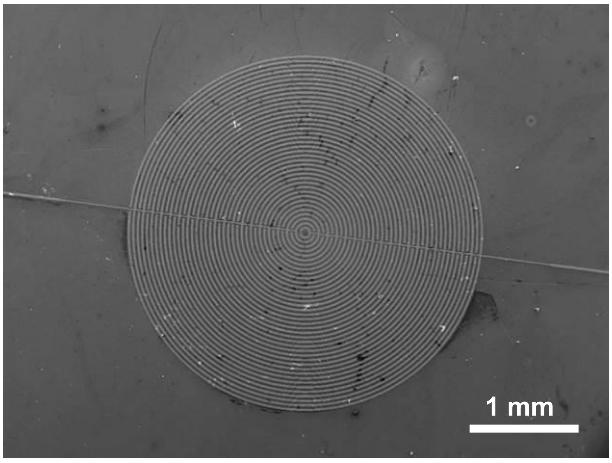

Figure S16. SEM image of Au-coated concentric interdigital microelectrode fabrication by photolithography.

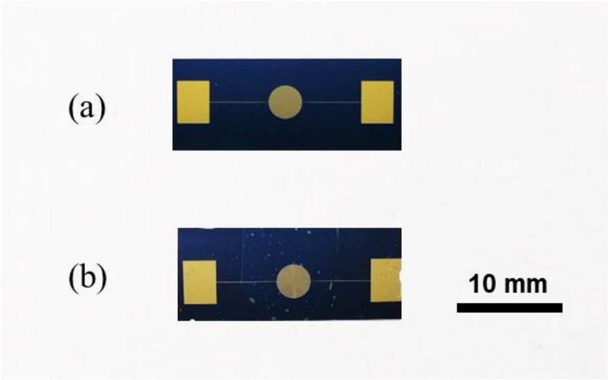

Figure S17. (a) Pristine Au-coated concentric interdigital microelectrode. (b) COF-QA-4 modified concentric interdigital microelectrode by spin-coating.

7.2 Contact angle measurements

The water contact angle value was reduced from 99.21° to 85.55° after the spin-coating of the COF-QA-4 (**Figure S18**). The change of electrode surface from hydrophobic to hydrophilic proved the success of surface adhesion and hydrophilicity.

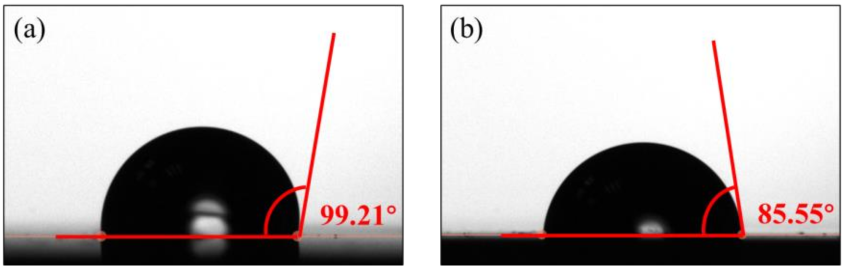

Figure S18. The contact angle of (a) pristine Au-coated concentric interdigital microelectrode. (b) COF-QA-4-modified concentric interdigital microelectrode by spin-coating method.

7.3 Calibration of EC gas concentrations

Because EC is in a solid state at room temperature, the thermogravimetric analysis (TGA) method was applied to calibrate the EC vapour concentrations under different N_2 supplying rates (**Table S11** and **Figure S19**) at the flow rate of 10 mL/min, 25 mL/min, 50 mL/min, 75 mL/min and 100 mL/min. By changing the flow rates of N_2, the different concentrations of EC gas can be obtained. The partial pressure of EC and N_2 gases could be calculated using the Ideal Gas Law,

$$pV = \frac{m}{M}RT \quad \text{..S(3)}$$

where p, V, m, and M are the partial pressure, volume, mass and relative molecular mass of the ideal gas, respectively. R is the ideal gas constant, and T is the thermodynamic temperature of the ideal gas. The concentration of EC gas (in ppmv) was equal to p_{EC}/p_{N_2}. The concentration of EC gas corresponding to an N_2 flow rate of 50 mL/min is 1.15 ppmv, calculated according to equation S(3) in Supporting Information.

Table S11. Calculated concentrations of EC vapour under different N_2 supplying rates

Mass loss of EC (mg)	N_2 flow rate (mL/min)	Concentration of EC (ppmv)
0.0071	10	1.97
0.0159	25	1.47
0.0248	50	1.15
0.0268	75	0.827
0.0093	100	0.215

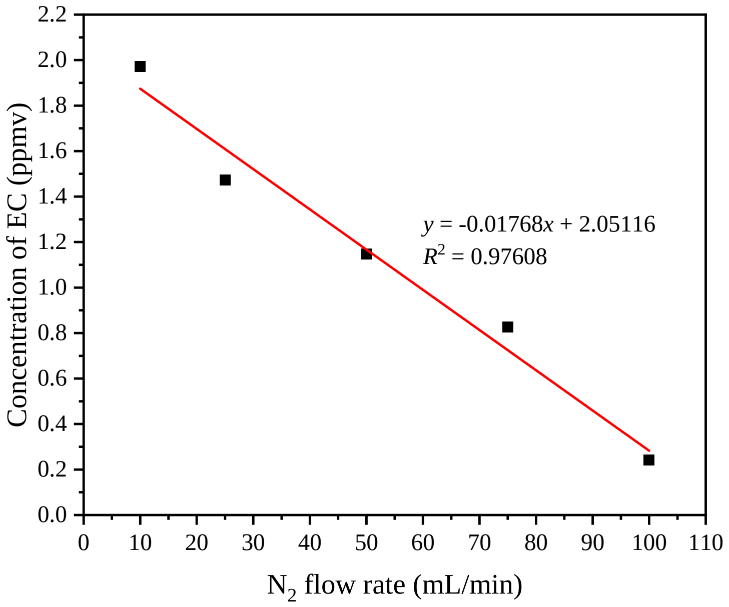

Figure S19. The relationship between EC vapour concentrations and N_2 flow rates.

7.4 The gas-sensing system

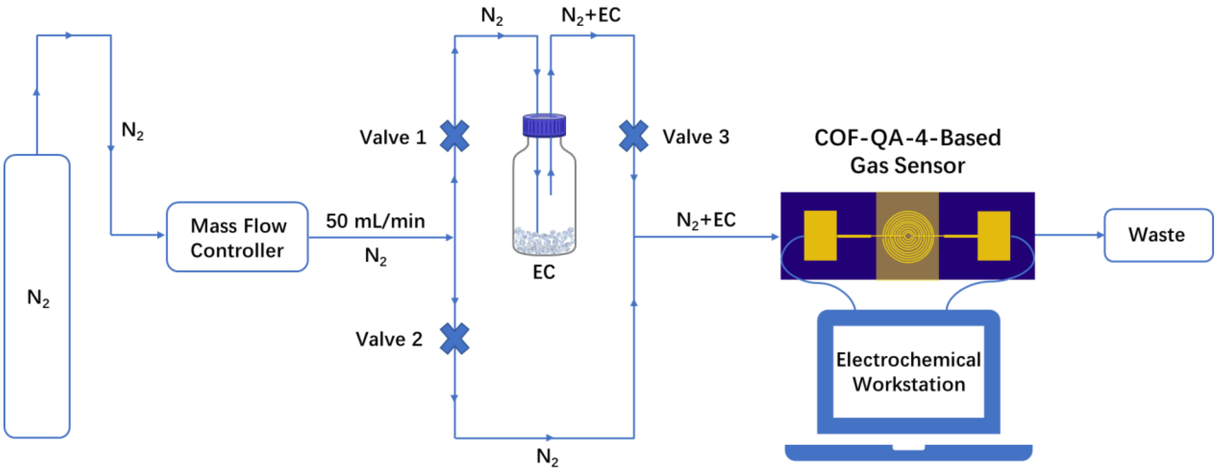

Figure S20. The scheme of the gas sensing system.

Table S12. Summary of the recent advances in gas-sensor for organic carbonate sensing

Materials	Target analyte	Detection limit	Reference
Cu-TCPP MOF	DMC	50 ppb	Ref.28
Double-walled carbon nanotubes	DMC	0.1 μL	Ref.29
SnO_2 Nanobox	EMC	20 ppb	Ref.30

Acronyms: Dimethyl carbonate (DMC), diethyl carbonate (DEC).

7.5 Adsorption capacity test

The microcantilever was employed to test the adsorption capacity of the synthesized COF-QA-4. Microcantilever has been useful in evaluating trace gas adsorption in sensing materials, such as volatile organic compounds (VOCs) gases.[31-33] Before the gas sensing test, the microcantilever was put into a 9.4 mL testing chamber to obtain a stable baseline signal. When the EC gases were introduced to the testing chamber, the adsorption of gas molecules in the sensing material coating on a microcantilever induced vibrational frequency changes and deflection. The gas flow rate was stabilized at 50 mL/min, and the temperature was 298 K. The frequency change was monitored through frequency−time measurements using an intelligent physicochemical parameters analyser (IPPA). The frequency changes due to the gases adsorbed in the sensing material can be converted to the change of mass after the gas adsorption using the following equation:

$$\Delta m = \frac{\Delta f}{S}$$

where Δm is the uptake of the gases, Δf is the frequency change after the gas adsorption. S is the sensitivity constant of the microcantilever, where S=0.24 Hz/pg for the applied microcantilever.

Based on the experimental results, the instantaneous uptake of EC gas with a partial pressure of 1.15 ppmv was 0.514 mg/g (**Figure S21**). This experimental adsorption capacity is much lower than the calculated uptake of 1 ppmv EC gas using GCMC simulation (5.88 mmol/g framework, 517.534 mg/g framework). The reason for the significant difference between the experimental and simulated results is that the removal of the solvent may cause the collapse and destruction of pore structures in the COF activation process, which leads to inefficient adsorption sites in the COF. This is supported by the reported BET surface area of the COF-QA-4 film (18 m^2/g), which is substantially lower than the calculated accessible surface area of 4854.48 m^2/g, obtained using Zeo++ software. Despite this

difference, the sensing experimental results confirm that COF-QA-4 exhibits excellent sensing signals toward trace amounts of EC gas.

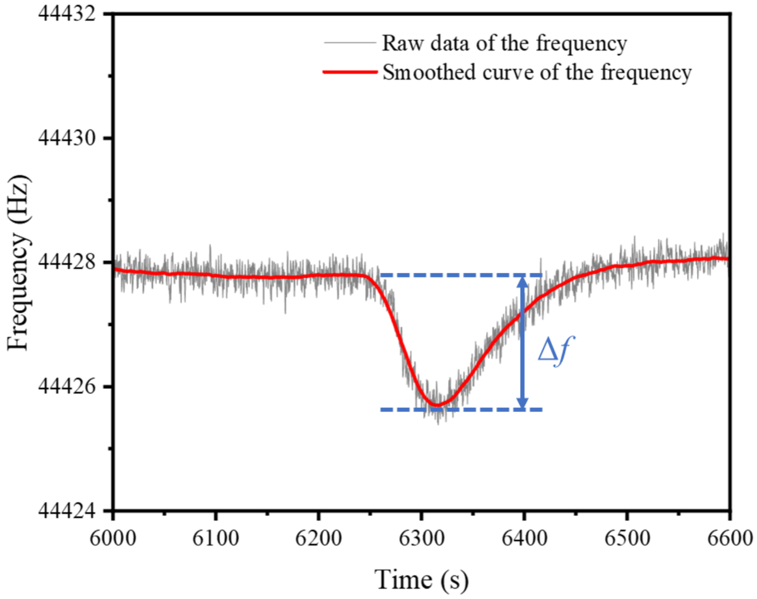

Figure S21. The measured change of frequency versus time after the EC gas adsorption in the COF-QA-4 using the microcantilever.

8. Characterization of monomer

8.1 NMR analysis of monomer

The ^{1}H and ^{13}C NMR spectra of the product (P1-P3) in the multi-step reaction were characterized respectively by Bruker Ascend 400 NMR spectrometer (400 MHz for ^{1}H NMR, 100 MHz for ^{13}C NMR).

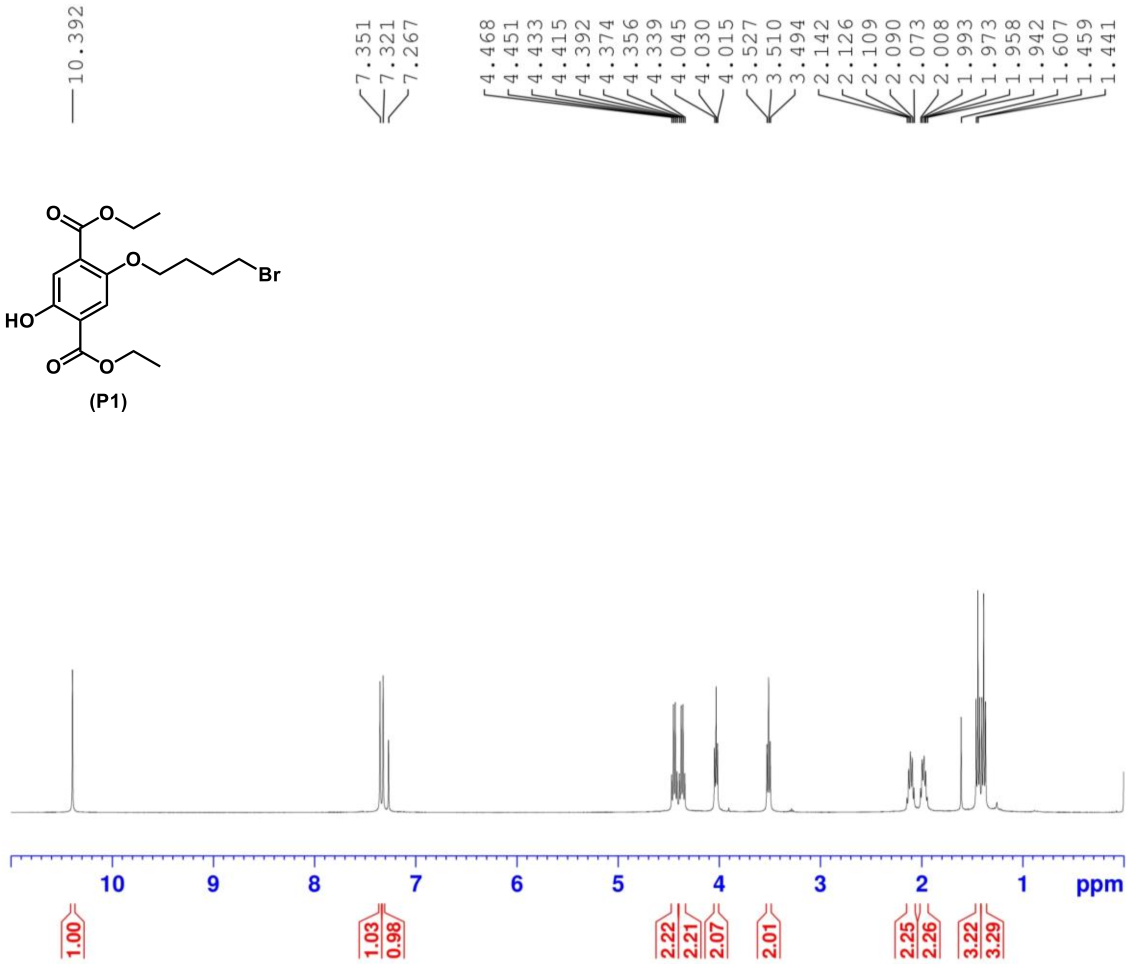

Figure S22. ^{1}H NMR (400 MHz, 298 K, $CDCl_3$) of **P1**: δ = 10.38 (s, 1H), 7.35 (s, 1H), 7.32 (s, 1H), 4.45 (q, J = 7.2 Hz, 2H), 4.37 (q, J = 7.2 Hz, 2H), 4.03 (t, J = 6.0 Hz, 2H), 3.51 (t, J = 6.8 Hz, 2H), 2.11 (p, J = 6.8 Hz, 2H), 1.99 (p, J = 8.0 Hz, 2H), 1.44 (t, J = 7.2 Hz, 3H), 1.38 (t, J = 7.2 Hz, 3H).

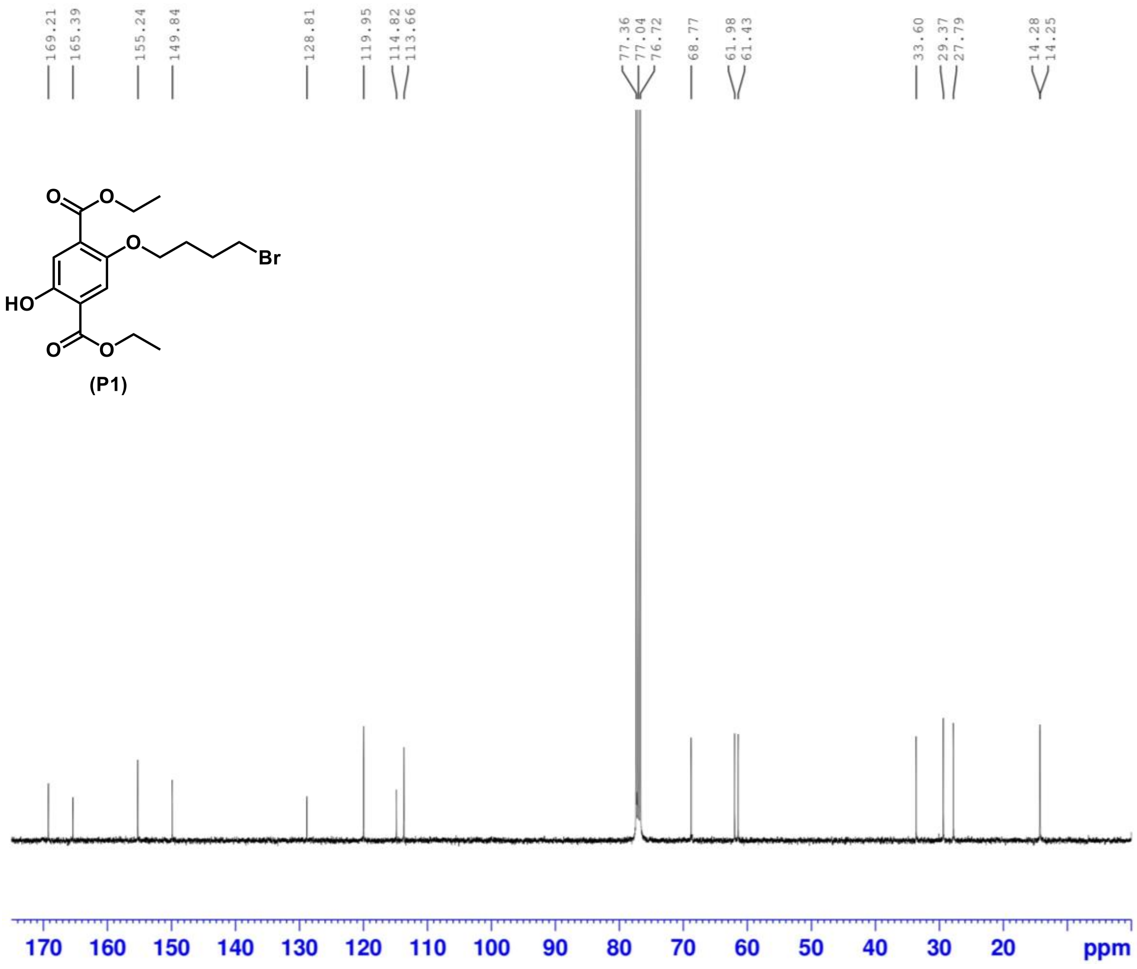

Figure S23. ^{13}C NMR (100 MHz, 298 K, $CDCl_3$) of the **P1**: δ = 169.21, 165.39, 155.24, 149.84, 128.81, 119.95, 114.82, 113.66, 68.77, 61.98, 61.43, 33.60, 29.37, 27.79, 14.28, 14.25.

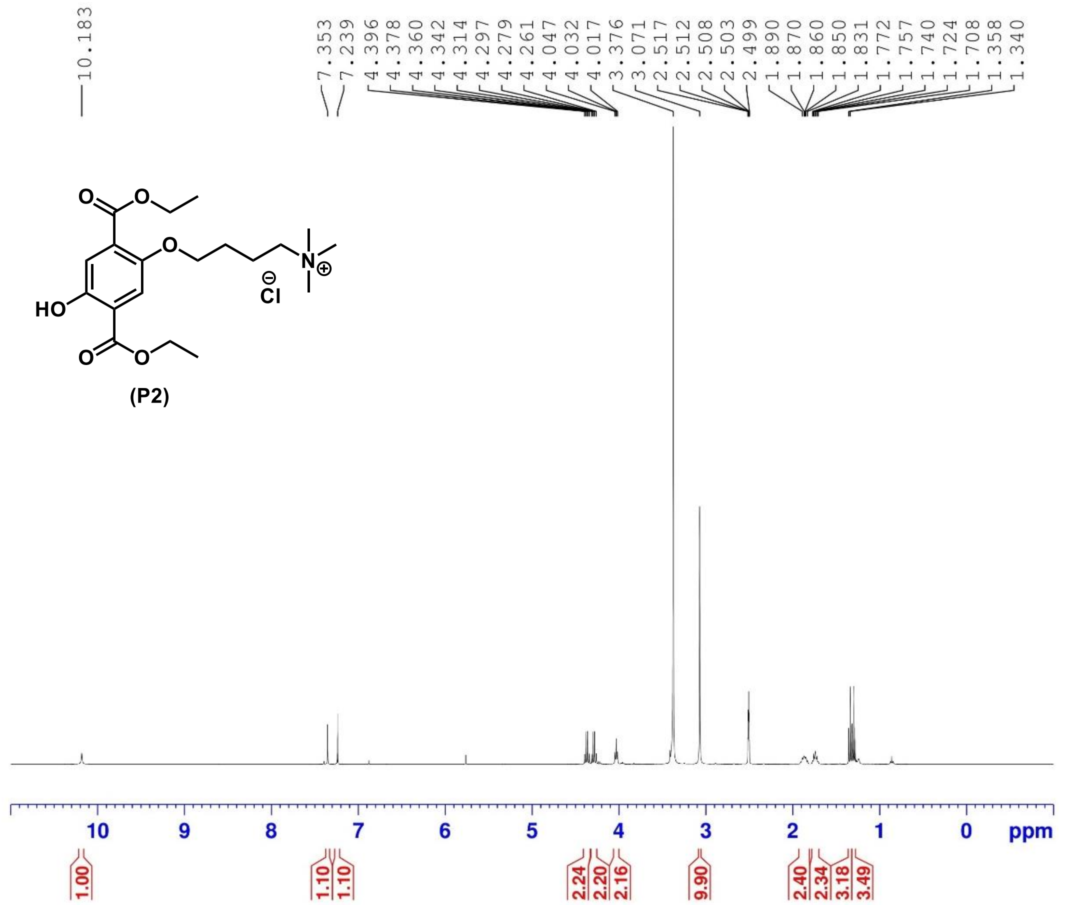

Figure S24. ^{1}H NMR (400 MHz, 298 K, DMSO-d_6) of **P2**: δ = 10.18 (s, 1H), 7.35 (s, 1H), 7.24 (s, 1H), 4.36 (q, J = 7.2 Hz, 2H), 4.28 (q, J = 7.2 Hz, 2H), 4.03 (t, J = 6.0 Hz, 2H), 3.43-3.37 (m, 2H), 3.07 (s, 9H), 1.93-1.81 (m, 2H), 1.74 (p, J = 6.8 Hz, 2H), 1.34 (t, J = 7.2 Hz, 3H), 1.30 (t, J = 7.2 Hz, 3H).

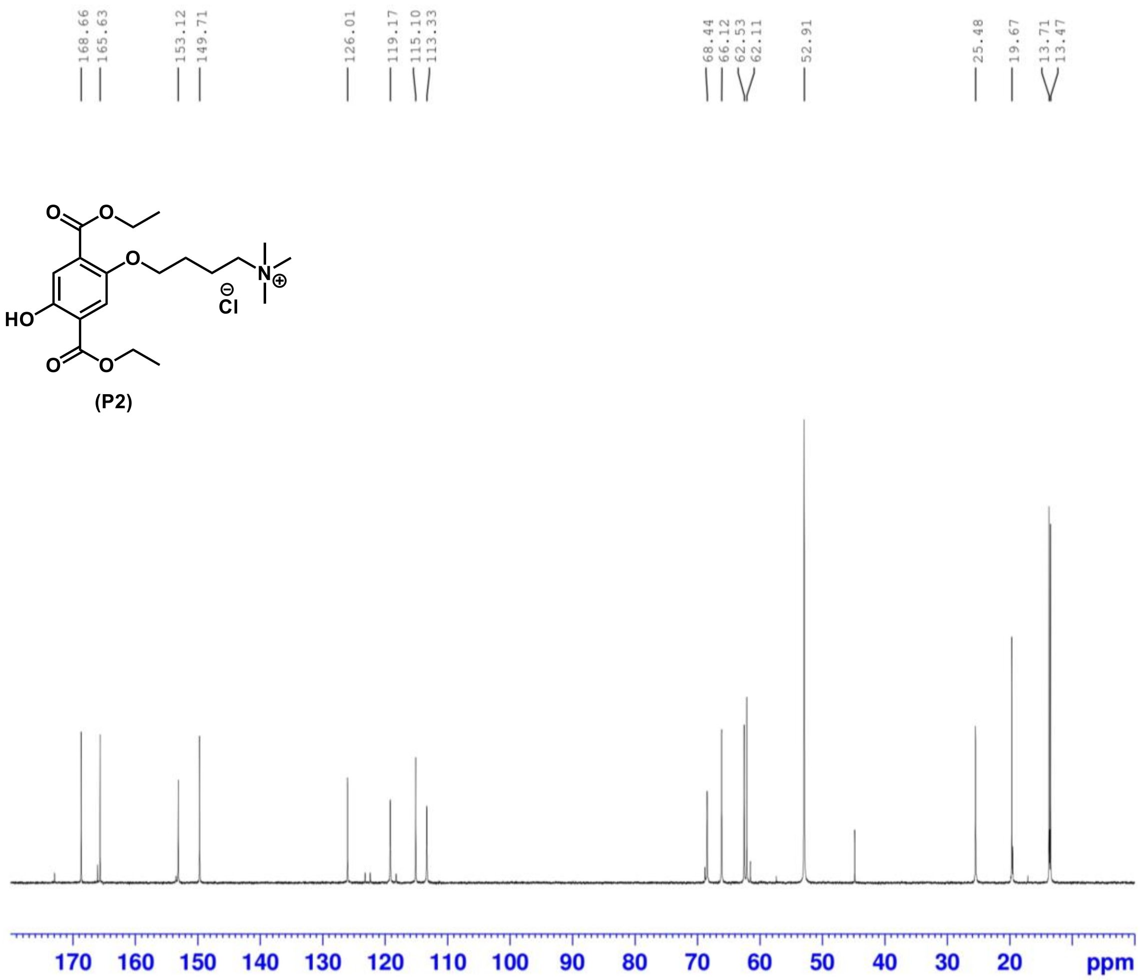

Figure S25. ^{13}C NMR (100 MHz, 298 K, D_2O) of **P2**: δ = 168.66, 165.63, 153.12, 149.71, 126.01, 119.17, 115.10, 113.33, 68.44, 66.12, 62.53, 62.11, 52.91, 25.48, 19.67, 13.71, 13.47.

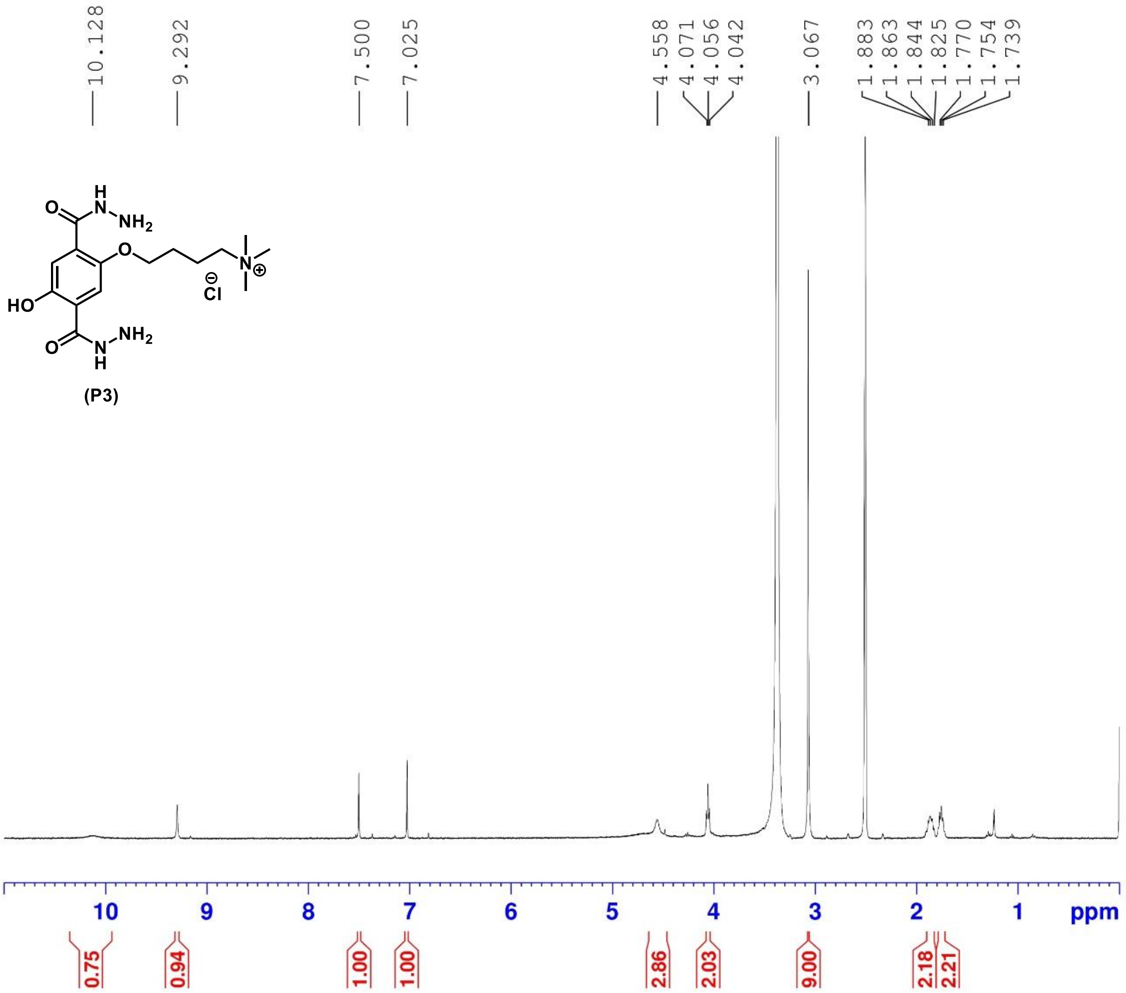

Figure S26. ^{1}H NMR (400 MHz, 298 K, DMSO-d_6) of **P3**: δ = 10.13 (s, 1H), 9.30 (s, 1H), 7.50 (s, 1H), 7.03 (s, 1H), 4.56 (s, 3H), 4.06 (t, J = 6.0 Hz, 2H), 3.40-3.35 (m, 2H), 3.07 (s, 9H), 1.95-1.80 (m, 2H), 1.75 (p, J = 7.2, 6.4 Hz, 2H).

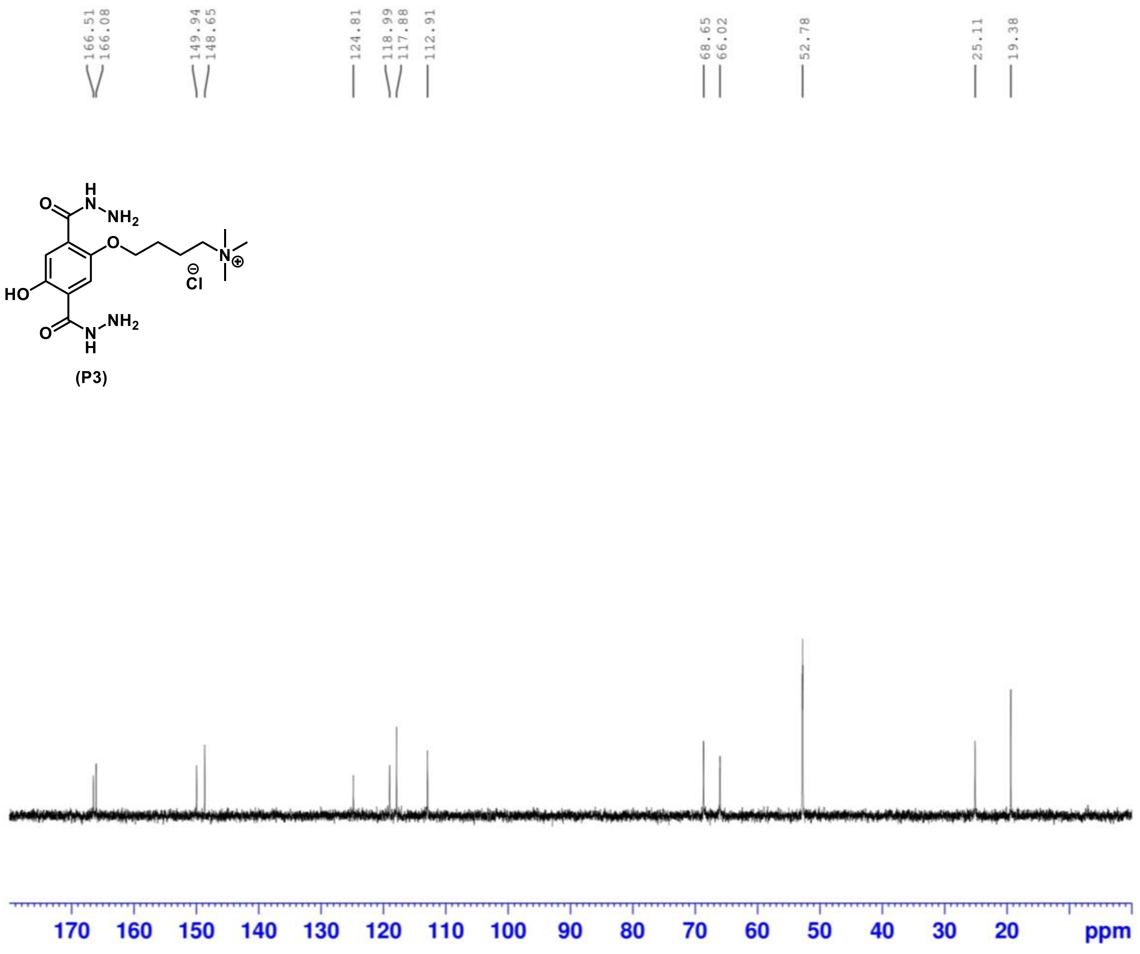

Figure S27. ^{13}C NMR (100 MHz, 298 K, D_2O) of **P3**: δ = 166.51, 166.08, 149.94, 148.65, 124.81, 118.99, 117.88, 112.91, 68.65, 66.02, 52.78, 25.11, 19.38.

8.2 FTIR spectroscopy of monomer

The chemical compositions of the 2,5-dihydroxyterephthalic acid diethyl ester (starting material), P1, P2 and P3 (QA-4 functionalized group) were characterized by FTIR spectroscopy. In **Figure S28**, the disappearance of stretching vibrations of C=O and O–C–O at 1330 ~ 1050 cm^{-1} from the ester and the appearance of O=C–N at 1680 ~ 1630 cm^{-1} from the secondary amide indicated that the P3 (QA-4 functionalized group) had been successfully synthesized.

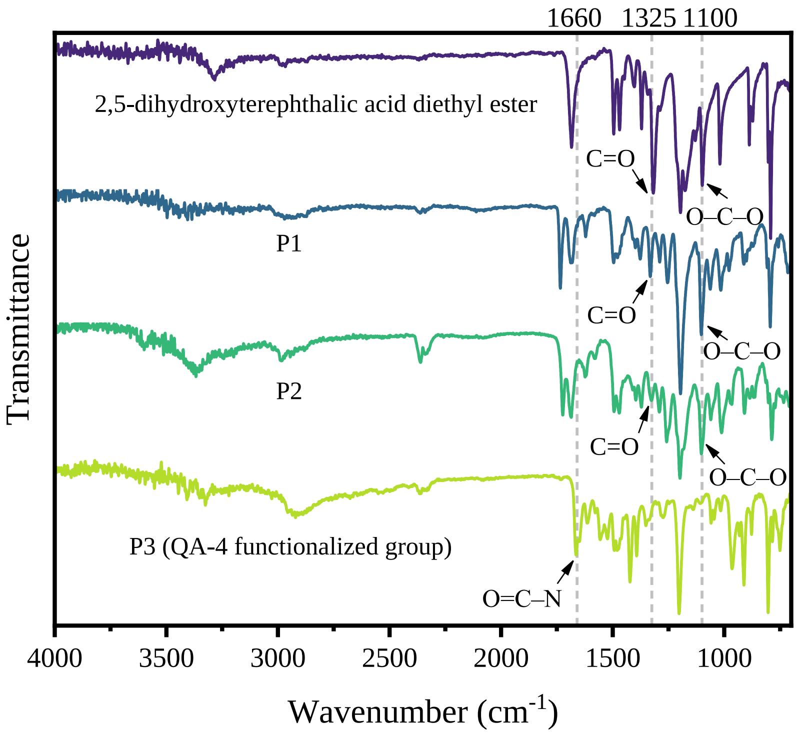

Figure S28. FTIR spectra of 2,5-dihydroxyterephthalic acid diethyl ester (starting material), P1, P2 and P3 (QA-4 functionalized group).

8.3 Mass Spectra

The mass spectra of the QA-4 functionalized group were characterized in positive ion mode via liquid chromatography quadrupole time-of-flight mass spectrometry (LC-Q-TOF-MS) in positive ion mode using full scan acquisition (**Figure S29**). The result showed the presence of fragments with m/z 340.1754, which matches exactly with the molecule weight of the $[M]^+$ of the QA-4 functionalized group at 340.1945. It only has a difference of 0.0056% compared to the standard COF-QA-4 molecular weight, which is acceptable for such an accurate checking. It indicates that COF-QA-4 has been successfully synthesized by the multi-step reaction mentioned in **Supporting Information 5.1**.

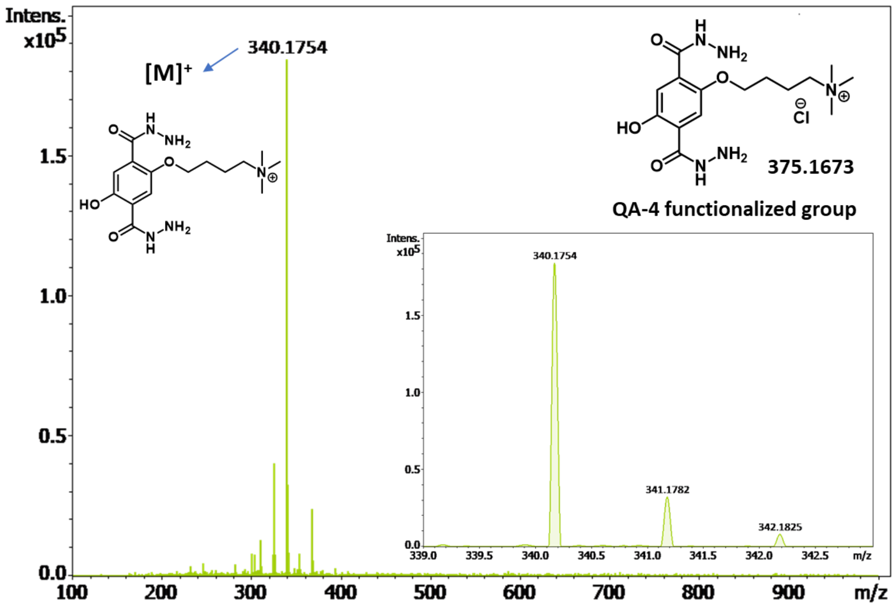

Figure S29. Fragmentation mass spectra of QA-4 functionalized group by LC-Q-TOF-MS acquired in positive ion mode.

9. Reference

(1) Masia, M.; Probst, M.; Rey, R. Ethylene Carbonate−Li^+: A Theoretical Study of Structural and Vibrational Properties in Gas and Liquid Phases. *The Journal of Physical Chemistry B* **2004**, *108*, 2016–2027.

(2) Webster, C. E.; Drago, R. S.; Zerner, M. C. Molecular Dimensions for Adsorptives. *Journal of the American Chemical Society* **1998**, *120*, 5509–5516.

(3) Petitjean, M. About the Algebraic Solutions of Smallest Enclosing Cylinders Problems. *Applicable Algebra in Engineering, Communication and Computing* **2012**, *23*, 151–164.

(4) Soetens, J.-C.; Millot, C.; Maigret, B. Molecular Dynamics Simulation of $Li^+BF_4^-$ in Ethylene Carbonate, Propylene Carbonate, and Dimethyl Carbonate Solvents. *The Journal of Physical Chemistry A* **1998**, *102*, 1055–1061.

(5) Postupna, O. O.; Kolesnik, Y. V.; Kalugin, O. N.; Prezhdo, O. V. Microscopic Structure and Dynamics of $LiBF_4$ Solutions in Cyclic and Linear Carbonates. *The Journal of Physical Chemistry B* **2011**, *115*, 14563–14571.

(6) Silva, L. B.; Freitas, L. C. Structural and Thermodynamic Properties of Liquid Ethylene Carbonate and Propylene Carbonate by Monte Carlo Simulations. *Journal of Molecular Structure: THEOCHEM* **2007**, *806*, 23–34.

(7) Maerzke, K. A.; Schultz, N. E.; Ross, R. B.; Siepmann, J. I. TraPPE-UA Force Field for Acrylates and Monte Carlo Simulations for Their Mixtures with Alkanes and Alcohols. *The Journal of Physical Chemistry B* **2009**, *113*, 6415–6425.

(8) Stubbs, J. M.; Potoff, J. J.; Siepmann, J. I. Transferable Potentials for Phase Equilibria. 6. United-Atom Description for Ethers, Glycols, Ketones, and Aldehydes. *The Journal of Physical Chemistry*

B **2004**, *108*, 17596–17605.

(9) Sirjoosingh, A.; Alavi, S.; Woo, T. K. Grand-Canonical Monte Carlo and Molecular-Dynamics Simulations of Carbon-Dioxide and Carbon-Monoxide Adsorption in Zeolitic Imidazolate Framework Materials. *The Journal of Physical Chemistry C* **2010**, *114*, 2171–2178.

(10) Qiao, Z.; Zhang, K.; Jiang, J. In Silico Screening of 4764 Computation-Ready, Experimental Metal–Organic Frameworks for CO_2 Separation. *Journal of Materials Chemistry A* **2016**, *4*, 2105–2114.

(11) Berendsen, H. J.; Grigera, J. R.; Straatsma, T. P. The Missing Term in Effective Pair Potentials. *The Journal of Physical Chemistry* **1987**, *91*, 6269–6271.

(12) Vujić, B.; Lyubartsev, A. P. Transferable Force-Field for Modelling of CO_2, N_2, O_2 and Ar in All Silica and Na^+ Exchanged Zeolites. *Modelling and Simulation in Materials Science and Engineering* **2016**, *24*, 045002.

(13) Yang, W.; Liang, H.; Peng, F.; Liu, Z.; Liu, J.; Qiao, Z. Computational Screening of Metal–Organic Framework Membranes for the Separation of 15 Gas Mixtures. *Nanomaterials* **2019**, *9*, 467.

(14) Martin, M. G.; Siepmann, J. I. Transferable Potentials for Phase Equilibria. 1. United-Atom Description of *n*-Alkanes. *The Journal of Physical Chemistry B* **1998**, *102*, 2569–2577.

(15) Wick, C. D.; Martin, M. G.; Siepmann, J. I. Transferable Potentials for Phase Equilibria. 4. United-Atom Description of Linear and Branched Alkenes and Alkylbenzenes. *The Journal of Physical Chemistry B* **2000**, *104*, 8008–8016.

(16) Erbil, H. Y. *Surface Chemistry of Solid and Liquid Interfaces*; John Wiley & Sons, 2006.

(17) He, X.; Yang, Y.; Wu, H.; He, G.; Xu, Z.; Kong, Y.; Cao, L.; Shi, B.; Zhang, Z.; Tongsh, C.; *et al.* De Novo Design of Covalent Organic Framework Membranes toward Ultrafast Anion Transport.

Advanced Materials **2020**, *32*, 2001284.

(18) Morgan, P. W.; Kwolek, S. L. Interfacial Polycondensation. II. Fundamentals of Polymer Formation at Liquid Interfaces. *Journal of Polymer Science* **1959**, *40*, 299–327.

(19) Yuan, S.; Gu, J.; Zheng, Y.; Jiang, W.; Liang, B.; Pehkonen, S. O. Purification of Phenol-Contaminated Water by Adsorption with Quaternized Poly(Dimethylaminopropyl Methacrylamide)-Grafted PVBC Microspheres. *Journal of Materials Chemistry A* **2015**, *3*, 4620–4636.

(20) Ech-chamikh, E.; Essafti, A.; Ijdiyaou, Y.; Azizan, M. XPS Study of Amorphous Carbon Nitride (A-C:N) Thin Films Deposited by Reactive RF Sputtering. *Solar Energy Materials and Solar Cells* **2006**, *90*, 1420–1423.

(21) Zhang, T.; Qian, C.; Guo, P.; Gan, S.; Dong, L.; Bai, G.; Guo, Q. A Novel Reduced Graphene Oxide-Attapulgite (RGO-ATP) Supported Fe_2O_3 Catalyst for Heterogeneous Fenton-like Oxidation of Ciprofloxacin: Degradation Mechanism and Pathway. *Catalysts* **2020**, *10*, 189.

(22) Olivares-Xometl, O.; Likhanova, N. V.; Domínguez-Aguilar, M. A.; Hallen, J. M.; Zamudio, L. S.; Arce, E. Surface Analysis of Inhibitor Films Formed by Imidazolines and Amides on Mild Steel in an Acidic Environment. *Applied Surface Science* **2006**, *252*, 2139–2152.

(23) Chen, F.; Wu, Y.; Ning, J.; Ren, J.; Zhang, Z.; Zheng, C.; Zhong, Y.; Hu, Y. Facile Preparation of Ternary Ag_2CO_3/Ag/PANI Composite Nanorods with Enhanced Photoactivity and Stability. *Journal of Materials Science* **2017**, *52*, 4521–4531.

(24) Majumdar, A.; Das, S. C.; Shripathi, T.; Hippler, R. Chemical Synthesis and Surface Morphology of Amorphous Hydrogenated Carbon Nitride Film Deposited by N_2/CH_4 Dielectric Barrier Discharge Plasma. *Composite Interfaces* **2012**, *19*, 161–170.

(25) Afshari, M.; Dinari, M.; Farrokhpour, H.; Zamora, F. Imine-Linked Covalent Organic Framework with a Naphthalene Moiety as a Sensitive Phosphate Ion Sensing. *ACS Applied Materials & Interfaces* **2022**, *14*, 22398–22406.

(26) Song, X.-Z.; Zhao, Y.-H.; Zhang, F.; Ni, J.-C.; Zhang, Z.; Tan, Z.; Wang, X.-F.; Li, Y. Coupling Plant Polyphenol Coordination Assembly with $C_O(OH)_2$ to Enhance Electrocatalytic Performance towards Oxygen Evolution Reaction. *Nanomaterials* **2022**, *12*, 3972.

(27) Wilson, M.; Kore, R.; Ritchie, A. W.; Fraser, R. C.; Beaumont, S. K.; Srivastava, R.; Badyal, J. P. S. Palladium–Poly(Ionic Liquid) Membranes for Permselective Sonochemical Flow Catalysis. *Colloids and Surfaces A: Physicochemical and Engineering Aspects* **2018**, *545*, 78–85.

(28) Lu, Y.; Zhang, S.; Dai, S.; Liu, D.; Wang, X.; Tang, W.; Guo, X.; Duan, J.; Luo, W.; Yang, B.; *et al.* Ultrasensitive Detection of Electrolyte Leakage from Lithium-Ion Batteries by Ionically Conductive Metal-Organic Frameworks. *Matter* **2020**, *3*, 904–919.

(29) Du, X.; Yang, B.; Lu, Y.; Guo, X.; Zu, G.; Huang, J. Detection of Electrolyte Leakage from Lithium-Ion Batteries Using a Miniaturized Sensor Based on Functionalized Double-Walled Carbon Nanotubes. *Journal of Materials Chemistry C* **2021**, *9*, 6760–6765.

(30) Su, H.; Yang, H.; Ma, C.; Tang, J.; Zhu, C.; Wang, X.; Zeng, D. High Response and Selectivity of the SnO_2 Nanobox Gas Sensor for Ethyl Methyl Carbonate Leakage Detection in a Lithium-Ion Battery. *ACS Sensors* **2024**, *9*, 444–454.

(31) Bao, Y.; Xu, P.; Cai, S.; Yu, H.; Li, X. Detection of Volatile-Organic-Compounds (VOCs) in Solution Using Cantilever-Based Gas Sensors. *Talanta* **2018**, *182*, 148–155.

(32) Jahangir, I.; Koley, G. Dual-Channel Microcantilever Heaters for Volatile Organic Compound Detection and Mixture Analysis. *Scientific Reports* **2016**, *6*(1), 28735.

(33) Dong, Y.; Gao, W.; Zhou, Q.; Zheng, Y.; You, Z. Characterization of the Gas Sensors Based on Polymer-Coated Resonant Microcantilevers for the Detection of Volatile Organic Compounds. *Analytica Chimica Acta* **2010**, *671*, 85–91.
